# Recent Advances on the Role of Brain-Derived Neurotrophic Factor (BDNF) in Neurodegenerative Diseases

**DOI:** 10.3390/ijms23126827

**Published:** 2022-06-19

**Authors:** Khairunnuur Fairuz Azman, Rahimah Zakaria

**Affiliations:** Department of Physiology, School of Medical Sciences, Universiti Sains Malaysia, Kubang Kerian 16150, Kelantan, Malaysia; rahimah@usm.my

**Keywords:** BDNF, neurodegeneration, Alzheimer, Parkinson, Huntington, amyotrophic lateral sclerosis

## Abstract

Neurotrophins, such as brain-derived neurotrophic factor (BDNF), are essential for neuronal survival and growth. The signaling cascades initiated by BDNF and its receptor are the key regulators of synaptic plasticity, which plays important role in learning and memory formation. Changes in BDNF levels and signaling pathways have been identified in several neurodegenerative diseases, including Alzheimer’s disease, Parkinson’s disease, and Huntington’s disease, and have been linked with the symptoms and course of these diseases. This review summarizes the current understanding of the role of BDNF in several neurodegenerative diseases, as well as the underlying molecular mechanism. The therapeutic potential of BDNF treatment is also discussed, in the hope of discovering new avenues for the treatment of neurodegenerative diseases.

## 1. Introduction

Neurodegenerative diseases are characterized by the gradual loss of neuronal structure or function, which can lead to neuronal death. Neurodegenerative disease often results in progressive cognitive, functional, and behavioral changes, manifested as dysfunctional motor and cognitive impairment. The most common types of neurodegenerative disease, such as Alzheimer’s disease, Parkinson’s disease, Huntington’s disease, and amyotrophic lateral sclerosis (ALS), have all been extensively studied. Neurodegenerative diseases represent a major socioeconomic burden worldwide. The risk of acquiring a neurodegenerative disease increases dramatically with age [1]. Thus, with an aging population, the number of people affected is set to increase even further, necessitating the development of therapeutic strategies capable of reversing or stopping the degenerative process.

Neurotrophins are regulatory factors that mediate the differentiation and survival of neurons [2]. Neurotrophins include nerve growth factor (NGF), brain-derived neurotrophic factor (BDNF), neurotrophin-3 (NT3), and neurotrophin-4/5 (NT4/5), which are all derived from a common ancestral gene and have similar sequences and structures. Among neurotrophins, BDNF has been extensively investigated, and it has emerged as an important regulator of synaptic plasticity, neuronal survival, and differentiation. In addition, BDNF serves as a crucial molecular target for the development of drugs to treat neurological diseases. The first evidence of BDNF’s role in the etiology of neurodegenerative illnesses was discovered in the early 1990s. Since then, research on BDNF in neurodegenerative diseases has grown tremendously. In this review, we present recent updates on the role of BDNF and its downstream signaling pathways in neurodegenerative diseases, including Alzheimer’s disease, Parkinson’s disease, Huntington’s disease, and ALS. In addition, the therapeutic potential of BDNF in the treatment of neurodegenerative diseases will be reviewed. Scopus database was searched for articles published between 2010 and 2022. The search terms included: “BDNF”, “neurodegenerative diseases”, “Alzheimer’s disease”, “Parkinson’s disease”, “Huntington’s disease”, and “amyotrophic lateral sclerosis”. Full articles were obtained and the references were reviewed for additional information when necessary.

## 2. BDNF Molecular Mechanisms and Signaling Cascades

**Figure 1:**
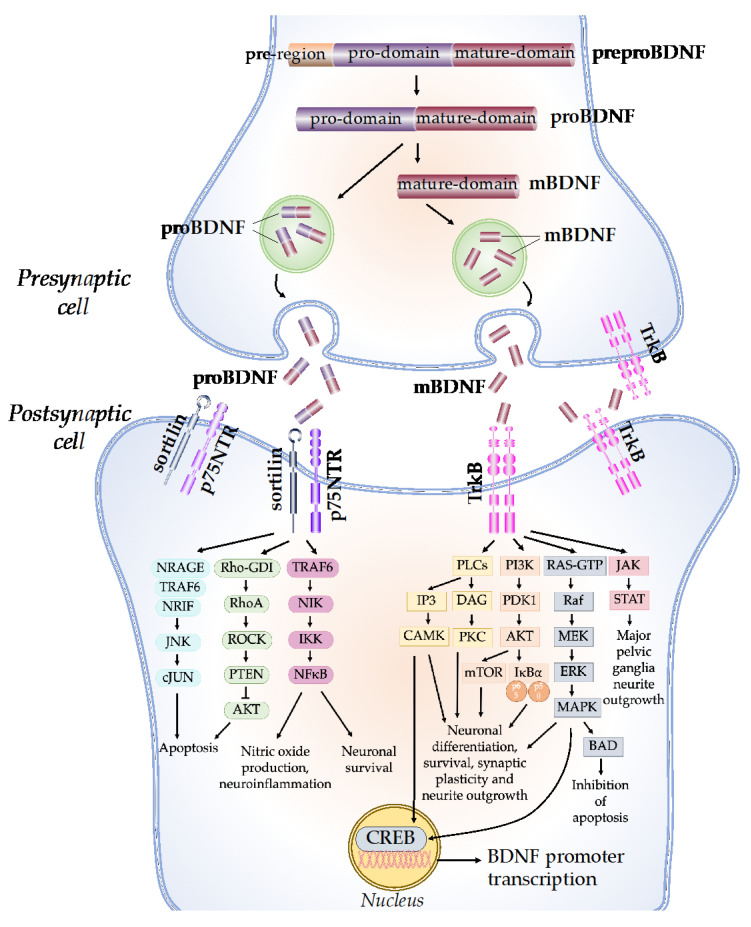
BDNF molecular mechanisms and signaling cascades. The BDNF protein is synthesized as a precursor form, preproBDNF. PreproBDNF is then converted into proBDNF, which consists of a pro-domain and a mature domain. The proBDNF is further cleaved to generate the mature isoform, mBDNF. ProBDNF and mBDNF are exocytosed into the extracellular space. The pro-domain of the proBDNF binds to the sortilin receptor, while the mature domain binds to the p75NTR receptor and activates the JNK/cJUN, PI3K/AKT, and TRAF6/NF-kB signaling pathways, which determine neuronal fate. The mBDNF binds to the TrkB receptor and activates PLCs, PI3K, MAPK, and JAK/STAT signaling cascades, which promote CREB translation, neuronal survival, and synaptic plasticity.

The synthesis and maturation of BDNF is a multistep process, starting with the formation of several precursor isoforms. The precursor form of BDNF protein, preproBDNF, is synthesized in the endoplasmic reticulum (Figure 1) [3]. PreproBDNF is then converted into a precursor proneurotrophin isoform of BDNF (proBDNF) by the removal of the signal peptide. The proBDNF is made up of 129 amino acids with an N-terminal pro-domain and 118 amino acids, with a mature domain at the C-terminus [4]. The mature isoform of the BDNF (mBDNF) is produced by additional cleavage of the proBDNF. ProBDNF can be cleaved intracellularly by endoproteases from the subtilisin-kexin family, such as furin, or by convertases in intracellular vesicles [5]. Plasmin and matrix metalloproteases 2 and 9 (MMP2 and MMP9) are required for the extracellular cleavage of proBDNF [6]. The physiological activity of mBDNF and proBDNF is enabled by their secretion into the extracellular space. BDNF secretion can be either constitutive or activity-dependent, depending on the cell type [4]. In neuronal cells, both proBDNF and mBDNF are released upon cellular membrane depolarization [7]. The ratio of proBDNF to mBDNF varies, depending on the particular stages and regions of brain development. ProBDNF is higher in the early postnatal period, when it is important for the development of brain function, while mBDNF is higher in adulthood for brain function, such as neuroprotection and synaptic plasticity [8].

The network of BDNF/tyrosine receptor kinase B (TrkB) and BDNF/ p75 pan-neurotrophin receptor (p75NTR) signaling pathways was first graphically mapped by Sandhya et al. in 2013. Briefly, the mature domain of proBDNF interacts preferentially with the p75NTR, while the pro-domain interacts with the sortilin receptor or other vacuolar protein-sorting 10 protein (Vps10p) [9]. The binding of proBDNF to its specific receptor activates signaling pathways that determine the fate of neurons and synapses. The proBDNF/p75NTR/sortilin binding complex can cause the activation of the c-Jun amino-terminal kinase (JNK) pathway, leading to dendritic spine loss, caspase release, and neuronal apoptosis [10]. The activation of JNK requires neurotrophin receptor-interacting MAGE homolog (NRAGE), neurotrophin receptor-interacting factor (NRIF), and tumor necrosis factor receptor-associated factor 6 (TRAF6). The binding of proBDNF to p75NTR can also cause the activation of the RhoA/Rho-associated kinase (ROCK) signaling pathway [11]. ROCK then activates the phosphatase and tension homolog (PTEN), which blocks the phosphoinositide 3-kinases-protein kinase B (PI3K/AKT) signaling necessary for TrkB-induced potentiation, thus eliciting apoptosis [12]. In addition, the TRAF6 signaling pathway is initiated, which leads to nuclear factor kappa B (NF-kB) activation. The activation of NF-kB can either promotes neuronal survival or nitric oxide production and neuroinflammation through multiple reactions [13].

On the other hand, mBDNF binds with the TrkB receptor, which has a high affinity. Upon BDNF binding, TrkB dimerizes, intracellular tyrosine residues are autophosphorylated, and several enzymes are activated, including phospholipase C (PLC), PI3K, guanosine triphosphate hydrolases (GTP), and Janus kinase (JAK) [14]. Through the activation of calcium-calmodulin-dependent protein kinase (CAMK) and protein kinase C (PKC), the PLC-dependent pathway leads to calcium-dependent signaling steps and the release of calcium ions from intracellular calcium storage, resulting in increased synaptic plasticity [15]. The PI3K/AKT-related pathway modulates N-methyl-D-aspartate receptor (NMDAR)-dependent synaptic plasticity and exerts pro-survival and antiapoptotic activity [16]. The PI3K/Akt/mTOR cascade promotes dendritic growth and branching by regulating the synthesis of protein and the development of the cytoskeleton [17]. The mitogen-activated protein kinase (MAPK)/RAS-signaling cascade regulates the synthesis of protein during neuronal development and is also involved in the activation of extracellular-signal-regulated kinase 1/2 (ERK 1/2) and cAMP response element-binding protein (CREB) [18]. CREB activation leads to the initiation of transcription, the prolongation of synaptic potentiation, dendritic arborization, and neuroprotection [19]. The JAK/STAT pathway promotes major pelvic ganglia neurite outgrowths [20].

The specialized role of BDNF in the regulation of numerous physiological brain processes is determined by the interaction of BDNF isoforms with different types of receptors. For example, the activation of TrkB by BDNF is important in the late phase of long-term potentiation (L-LTP), which stimulates structural changes at the synapse [21]. On the other hand, P75NTR activation by proBDNF facilitates hippocampal long-term depression (LTD), which results in a decrease in synaptic strength [21]. The disruptions in BDNF production that result in signaling-cascade failure may be responsible for a range of neurological disorders. In addition, changes in BDNF levels and activities have been associated with a variety of neurodegenerative diseases, including Alzheimer’s disease, Parkinson’s disease, Huntington’s disease, and ALS.

## 3. BDNF in Alzheimer’s Disease

Alzheimer’s disease is a progressive neurodegenerative disorder characterized by memory loss and multiple cognitive disorders. Currently, it is one of the ten most frequent causes of mortality worldwide, ranking third in the Americas and Europe in 2019 [22]. Women are disproportionately affected, accounting for 65 percent of Alzheimer’s disease deaths. Worldwide, around 45 million people have Alzheimer’s disease, and this number is projected to nearly triple by 2060 [23]. The consequences of Alzheimer’s disease have a substantial influence on individual lives and social expenditure, with treatment costs estimated to be as high as USD 600 billion per year [23]. Although considerable efforts have been undertaken to tackle it, Alzheimer’s disease remains incurable, mainly due to our poor knowledge about its complex pathological mechanism. The deposition of extracellular amyloid β (Aβ) protein, or ‘amyloid plaques’, in the brain is the first identifiable pathology, occurring decades before clinical symptoms appear [24]. The accumulation of Aβ contributes to the increased phosphorylation and secretion of Tau, a microtubule-associated axonal protein that is extensively expressed in cortical neurons [25]. Disruptions in tau metabolism cause neurodegeneration, resulting in dystrophic neurites and intraneuronal neurofibrillary tangles of hyperphosphorylated and truncated tau proteins. Neuronal and synaptic degeneration, as well as neurofibrillary tangles and neuropil threads, plaques, granulovacuolar degeneration, and Hirano bodies, are the most prominent microscopic neuropathological characteristics of Alzheimer’s disease [26]. Most Alzheimer’s disease cases are termed ‘sporadic’, with the common causes being a combination of hereditary and environmental risk factors. There are a few rare familial cases of Alzheimer’s disease, which are termed autosomal-dominant early-onset Alzheimer’s disease.

Several studies have underlined the link between Alzheimer’s disease and BDNF. The decreased BDNF protein and mRNA levels in the neocortex and hippocampus suggest that BDNF plays a significant role in Alzheimer’s disease [27,28,29,30]. Similarly, a significant decrease in BDNF levels in the peripheral blood of Alzheimer’s disease patients has been reported (Table 1) [31,32,33]. Despite this, several studies reported conflicting results, according to which there was an elevation in the BDNF levels in the peripheral blood of Alzheimer’s disease patients [34,35]. It was suggested that the increased BDNF levels were due to a compensatory mechanism to fight early neurodegeneration or to the activation of immune cells [34]. As the disease advances, these compensatory processes may begin to fail, resulting in lower BDNF levels in the peripheral blood. Nevertheless, several meta-analysis studies reported that peripheral BDNF levels decreased in Alzheimer’s disease [36,37,38]. A significant correlation between serum BDNF levels and medial temporal lobe atrophy has been reported; therefore, decreased serum BDNF can potentially be used as a biomarker for early Alzheimer’s disease detection [39].

The depletion of BDNF is associated with Aβ accumulation, Tau phosphorylation, neuroinflammation, and neuronal apoptosis [40]. Tau is involved in the Aβ-induced downregulation of BDNF; hence, Alzheimer’s disease treatments that focus solely on Aβ may be ineffective if the impact of Tau pathology on neurotrophic pathways is not considered [41]. In addition, BDNF/TrkB deficiency increases inflammatory cytokines and stimulates the JAK2/STAT3 pathway, resulting in transcription factor C/EBPβ overexpression. As a result, the expression of δ-secretase increases, causing δ-secretase to fragment APP and Tau, and subsequently death of neurons [40]. Oxidative stress and Aβ decrease PKCϵ expression, and reciprocally, a depression in PKCϵ reduces BDNF and MnSOD in hippocampal pyramidal neurons, resulting in oxidative stress and disrupting synaptic plasticity [42]. On the other hand, BDNF overexpression or BDNF gene delivery have been shown to attenuate behavioral deficits, reduced neuronal abnormality, alleviated synaptic degeneration, and prevented neuron loss, although they did not affect the Tau hyperphosphorylation level [43]. BDNF overexpression also improves the therapeutic potential of engrafted neural stem cells (NSCs) for Alzheimer’s disease via neuronal replacement and neurogenic effects, through which it improves the engrafted cells’ viability, neurite complexity, maturation of electrical properties, neuronal fate, and synaptic density [44]. Furthermore, conditional BDNF delivery from astrocytes has been shown to rescue memory deficits, spine density, and synaptic properties [45].

**Table 1:** Role of BDNF in Alzheimer’s disease.

Observations		References
Alzheimer’s disease patients		
Serum BDNF levels significantly decreased in early-onset and late-onset Alzheimer’s disease compared to age-matched healthy controls.		[32]
BDNF levels in platelet-rich plasma significantly decreased, which was correlated with moderate-to-severe stages of dementia.		[33]
Alzheimer’s disease patients have higher levels of peripheral BDNF, possibly due to a compensatory mechanism to fight early neurodegeneration or to the activation of immune cells.		[34]
BDNF serum levels are increased in subjects with MCI and decreased in subjects with Alzheimer’s disease.		[35]
There is a significant correlation between serum BDNF levels and medial temporal lobe atrophy.		[39]
Oxidative stress and Aβ decrease PKCϵ expression. A depression in PKCϵ reduces BDNF and MnSOD expression in hippocampal pyramidal neurons.		[42]
BDNF level was reduced in the sera and brains of Alzheimer’s disease patients.		[43]
Peripheral BDNF promoter methylation might be a diagnostic marker of Alzheimer’s disease risk.		[46]
There was a gender-related alteration in BDNF mRNA expression in brain tissues and a positive genetic association of rs6265 in BDNF with Alzheimer’s disease in females. There was a clear female-specific risk trend for the effect of BDNF rs6265 on Alzheimer’s disease-related endophenotypes.		[47]
The ApoE ε4 genotype is involved in regulating BDNF metabolism. The interaction between BDNF and ApoE genotype plays a critical role in Alzheimer’s disease pathogenesis.		[48]
There were dose-dependent genotype effects and significant correlations between the cognitive test scores and interconnected-cluster volumes, especially in the orbitofrontal cortex.		[49]
BDNF genetic variations increase the risk of Alzheimer’s-disease-related depression.		[50]
Prefrontal cortex BDNF gene expression is associated with aging, rs6265 carrier status, and AD neuropathology in a variant-specific manner that seems to be independent of DNA methylation influences.		[51]
BDNF-AS levels in the plasma of late-stage Alzheimer’s disease patients showed a significant increase compared to healthy subjects.		[52]
BDNF anti-sense RNA (BDNF-AS) promotes BACE1 expression and Alzheimer’s disease progression through the competitive binding of miR-9-5p.		[53]
Pro-BDNF levels are significantly associated with both amyloid load and pTau in the hippocampus.		[54]
Amnestic mild cognitive impairment (aMCI) patients		
The interactions between DNA methylation (CpG5) of the BDNF gene promoter and the tag SNP (rs6265) play important roles in the etiology of amnestic mild cognitive impairment (aMCI) and its conversion to Alzheimer’s disease.		[55]
The elevation of peripheral BDNF promoter methylation might be used as potential epigenetic biomarkers for predicting the conversion from aMCI to Alzheimer’s disease.		[56]
Patients with subjective cognitive decline (SCD) to mild cognitive impairment (MCI)		
BDNF Val66Met increased the risk of progression from SCD to MCI and from MCI to Alzheimer’s disease in women only.		[57]
Cognitively unimpaired (CU) adults		
The interaction between BDNF Met and APOE4 has a weak effect on amyloid-β plaque burden, and the longitudinal PET measurements of Alzheimer’s disease-related carriage have a weak effect on the decline in the cerebral metabolic rate for glucose (CMRgl) in cognitively unimpaired late-middle-aged and older adults, but there is no apparent effect on the rate of cognitive decline.		[58]
Animal model		
13.5-month-old BDNF^f/f^ and TrkB^f/f^ mice	The deprivation of BDNF/TrkB increases inflammatory cytokines and activates the JAK2/STAT3 pathway, resulting in the upregulation of transcription factor C/EBPβ. This, in turn, results in the increased expression of δ-secretase, leading to both APP and Tau fragmentation by δ-secretase and neuronal loss.	[40]
Transgenic mouse models of human Tau expression	Tau at least partially mediates Aβ-induced BDNF downregulation. Therefore, Alzheimer’s disease treatments targeting Aβ alone may not be effective without considering the impact of Tau pathology on neurotrophic pathways.	[41]
P301L transgenic mice (a mouse model of tauopathy)	The BDNF level was reduced in the sera and brains of P301L transgenic mice. BDNF overexpression attenuated behavioral deficits, prevented neuron loss, alleviated synaptic degeneration, and reduced neuronal abnormality, but did not affect Tau hyperphosphorylation levels.	[43]
5xFAD mouse model of Alzheimer’s disease	Conditional BDNF delivery from astrocytes rescues memory deficits, spine density, and synaptic properties.	[45]
APPswe/PS1dE9 (APdE9) mice	BDNF gene mutations are deleterious for learning and memory. BDNF protein accumulates around amyloid plaques in the brains of APdE9 mice.	[59]
Cell culture		
human Tau (hTau40)-transfected human neuroblastoma (SH-SY5Y) cells	Tau at least partially mediates Aβ-induced BDNF downregulation. Therefore, Alzheimer’s disease treatments targeting Aβ alone may not be effective without considering the impact of Tau pathology on neurotrophic pathways.	[41]
Beta-amyloid-treated neural stem cells (NSCs)	BDNF overexpression improves the therapeutic potential of engrafted NSCs for Alzheimer’s disease via neurogenic effects and neuronal replacement.	[44]
SH-SY5Y cell line	BDNF anti-sense RNA (BDNF-AS) promotes BACE1 expression and Alzheimer’s disease progression through the competitive binding of miR-9-5p.	[53]
SH-SY5Y cell line	There is a synergistic toxic interaction between the amyloid-β peptide (Aβ_1-42_) and the pro-domains of both DNT1 and BDNF.	[60]

In addition to the BDNF level, BDNF-promoter methylation in the peripheral blood has been investigated for the prediction of Alzheimer’s disease risk. It has been suggested that peripheral BDNF-promoter methylation has the potential to be a diagnostic marker of Alzheimer’s disease risk [46]. The interactions between the tag SNP (rs6265) and DNA methylation (CpG5) of the BDNF gene promoter are involved in the etiology of amnestic mild cognitive impairment (aMCI) and its conversion to Alzheimer’s disease [55]. Thus, the elevation of peripheral BDNF promoter methylation might be used as a potential epigenetic biomarker for the prediction of aMCI conversion to Alzheimer’s disease [56]. The risk of progression from MCI to Alzheimer’s disease is also associated with BDNF Val66Met polymorphism, whereby BDNF Val66Met increases the risk of disease progression [57]. The Met allele, on the other hand, only raised the risk of Alzheimer’s disease in women [57]. Similarly, there is a sex-related alteration in BDNF mRNA expression in brain tissues, as well as a positive genetic association of rs6265 in BDNF with Alzheimer’s disease in females. The influence of BDNF rs6265 on Alzheimer’s-disease-related endophenotypes had a significant female-specific risk trend [47].

In the pathogenesis of Alzheimer’s disease, the interplay between BDNF and the ApoE genotype is crucial [48]. There is a weak association between ApoE4 and BDNF Met on longitudinal PET measurements and the Aβ plaque burden of Alzheimer’s disease-related carriage on cerebral metabolic rate for glucose (CMRgl) decline in cognitively unimpaired late-middle-aged and older adults, without an obvious effect upon the rate of cognitive decline [58]. In addition, there are dose-dependent ApoE genotype effects and significant correlations between interconnected-cluster volumes and cognitive test scores, especially in the orbitofrontal cortex [49]. These discoveries support the hypothesis that BDNF rs6265 polymorphisms modulate entorhinal-cortex interconnected clusters [49]. Other studies have investigated the transcriptional, epigenetic, and BDNF translational regulation in the brain and its association with Alzheimer’s disease. BDNF gene mutations are deleterious to learning and memory [59]. In addition, the risk of Alzheimer’s-disease-related depression is increased with BDNF genetic variations [50]. Prefrontal-cortex BDNF gene expression is associated with rs6265 carrier status, aging, and Alzheimer’s disease neuropathology in a variant-specific pattern that appears to be distinct from DNA methylation [51].

A long noncoding RNA known as BDNF antisense (BDNF-AS) is one of the RNAs involved in Alzheimer’s disease. The plasma of Alzheimer’s disease patients showed a significant increase in BDNF-AS levels compared to those of healthy subjects [52]. BDNF-AS promotes BACE1 expression and Alzheimer’s disease progression through the competitive binding of miR-9-5p [53]. These findings suggest that the plasma levels of the long noncoding RNA BDNF-AS have the potential to be used as a blood/plasma diagnostic marker for Alzheimer’s disease diagnosis. The silencing of BDNF-AS RNA increases BDNF levels, enhances cell viability, and reduces Aβ-induced neurotoxicity [61].

In addition to BDNF, pro-BDNF has also been associated with Alzheimer’s disease and Tau. There is a synergistic toxic interaction between pro-BDNF and the Aβ peptide [60]. The activation of the BDNF pro-domain receptor p75NTR by Aβ1-42 is thought to be the cause of this synergistic interaction [60]. The pro-BDNF level has also been significantly associated with pTau and amyloid load in the hippocampus [54]. The ratio of BDNF pro-to-mature domains in Alzheimer’s disease patients’ brains was shown to increase by more than thirty-fold [60]. This imbalanced BDNF pro-to-mature-domain ratio in Alzheimer’s patients could be a biomarker for the disease.

In summary, the decreased BDNF levels in the blood and brains of Alzheimer’s disease patients suggest that BDNF is imperative in the etiology of Alzheimer’s disease and thus has the potential to be employed as a biomarker for the early detection of Alzheimer’s disease. BDNF depletion is associated with neuroinflammation, Tau phosphorylation, Aβ accumulation, and neuronal apoptosis. Interestingly, BDNF overexpression or gene delivery has been proven to alleviate neuronal abnormality, neuronal loss, synaptic degeneration, and behavioral deficits. Therefore, this evidence opens up a new avenue for the treatment of Alzheimer’s disease using BDNF therapy. However, the exact pathway implicated in the modulation of BDNF signaling warrants further investigation in order to precisely target the desired effect.

## 4. BDNF in Parkinson’s Disease

Parkinson’s disease is the second-worst neurodegenerative disease worldwide after Alzheimer’s disease. It affects 1-2 per 1000 of the population at any time [62]. The prevalence of Parkinson’s disease increases with age; 1% of the population over the age of 60 is affected. The disease costs over USD 51.9 billion annually in the US [63]. An increased understanding of this disease is critically important, particularly in countries with aging populations. The main neuropathological finding is the presence of α-synuclein-containing Lewy bodies and the loss of selected populations of dopaminergic neurons, particularly within the pars compacta of the substantia nigra (SNpc). In Parkinson’s disease, the axons of nigral dopamine (DA) neurons that transmit afferent axons to the striatum degenerate, causing striatal morphological alterations (e.g., altered synaptic connections, decreased spine density) and, ultimately, the loss of DA neurons in the midbrain, thus leading to both motor and non-motor symptoms. The classical features of Parkinson’s disease are resting-muscle rigidity, tremor, loss of postural reflexes, bradykinesia, flexed posture, mask-like facial expression, festinating gait, cognitive decline, handwriting changes, and postural deformities [64]. The causes of Parkinson’s disease are unknown, but substantial evidence suggests a multifactorial etiology involving environmental and genetic factors. Mutations in the gene encoding α-synuclein, deletions in the parkin gene, defects in mitochondrial metabolism, constitutive metabolic deficiencies, mitochondrial gene deletions, and oxidatively induced cellular damage have been associated with Parkinson’s disease etiology.

The established effects of BDNF on supporting the function and survival of substantia nigra (SN) DA neurons, as well as its structural and functional influence on striatal medium spiny neurons (MSNs), have sparked interest in the role of BDNF in Parkinson’s disease. BDNF has been shown to promote SN neuron survival in vitro and protects against various neurotoxic assaults in vitro and in vivo [65,66,67]. Furthermore, BDNF-TrkB signaling increases the number of docked vesicles within active zones at glutamatergic synapses, such as those established between cortical afferents and striatal MSNs, and also changes the activation kinetics of N-ethyl-D-aspartate (NMDA) and inhibitory gamma-amino butyric acid (GABA) receptors in the postsynaptic membrane [68]. Previous studies also showed that BDNF is essential for the function and maturation of these neurons and facilitates the establishment of striatal connections during brain development, the actin remodeling of MSNs, and dendritic spine dynamics [65,66,69,70].

Currently, there is strong evidence linking BDNF with Parkinson’s disease. For instance, several studies demonstrated a significant decrease in serum BDNF levels in patients with Parkinson’s disease (Table 2) [71,72,73,74,75,76]. These decreases in serum BDNF levels have been correlated with cognitive impairment [71,74], depression [72,76], and restless legs syndrome (RLS) [75]. In addition, lower levels of BDNF were significantly correlated with nigrostriatal system degeneration [73]. Moreover, the decreased peripheral alterations in BDNF/TrkB levels found in patients with Parkinson’s disease have been directly associated with the degeneration of dopaminergic neurons [77]. Recent evidence demonstrates that BDNF is strongly decreased in the guts and brains of Parkinson’s disease patients, and conditional BDNF knock-out in the gut elicits dopaminergic neuronal loss, motor dysfunctions, and constipation [78]. Gut inflammation exacerbates BDNF reduction by inducing C/EBPβ activation and triggers Parkinson’s disease non-motor and motor symptoms [78].

BDNF gene Val66Met polymorphism has been associated with an increased risk of Parkinson’s disease at an older age [79,80]. A later study, on the other hand, suggested that BDNF Val66Met polymorphism was not associated with Parkinson’s disease risk or onset, nor with cognitive status in Parkinson’s disease patients (Białecka et al., 2014). However, it was demonstrated that patients with Met/Met alleles exhibited better delayed recall of information than patients with Val/Val alleles [81]. These findings uncovered the possible difference in the implication of Parkinson’s disease progression between the genotypes. Val66Met (rs6265) is a common single-nucleotide polymorphism in the pro-domain of the BDNF protein that causes a valine (Val) to methionine (Met) substitution at amino acid position 66. The Met allele is heterozygous in approximately 20 to 30 percent of the human population. The presence of the minor allele (Met) of this polymorphism results in altered intracellular distribution and decreased activity-induced secretion of the BDNF protein in neurons [82]. Compared with Val homozygotes, the Met allele carriers demonstrated a higher prevalence of cognitive impairment in Parkinson’s disease patients [83]. The Met allele decreases neuronal dendrite distribution and targeting to secretory granules, lowering extracellular BDNF levels and causing cognitive impairment [83]. In addition, the Met allele has been associated with a higher neuropsychiatric burden in Parkinson’s disease [84]. However, one study suggests that carrying two copies of the Met allele is associated with a reduced severity of motor symptoms and, potentially, a slower rate of Parkinson’s disease progression [85]. Furthermore, the Met allele carriers demonstrated a significantly lower set-shifting decline compared with the homozygous Val allele carriers [86]. On the other hand, the G/G (Val/Val) genotype has been associated with depression and anxiety symptoms and the development of Parkinson’s disease [87]. The Val/Val genotype in Parkinson’s disease leads to a set of cortical and subcortical brain alterations that could increase cognitive decline in early Parkinson’s disease patients [88]. These disparities between BDNF genotypes and their effects on cognition, brain alterations, symptoms, and Parkinson’s disease progression warrants further research on the exact role of BDNF polymorphism in the pathomechanisms of Parkinson’s disease.

**Table 2:** Role of BDNF in Parkinson’s disease.

Observations		References
Parkinson’s disease patients		
Serum BDNF levels and cognitive function scores were significantly lower in Parkinson’s disease patients versus healthy controls.		[71]
Decreased serum BDNF may be involved in the pathophysiology of depression in Parkinson’s disease patients.		[72]
Serum BDNF levels were lower in recently diagnosed, untreated Parkinson’s disease patients compared to controls. These lower levels were significantly correlated with nigrostriatal system degeneration.		[73]
Low BDNF is associated with cognitive impairment in patients with Parkinson’s disease.		[74]
Decreased serum BDNF levels may be involved in the pathophysiology of restless legs syndrome (RLS) in Parkinson’s disease.		[75]
The serum BDNF levels were lower in depressed Parkinson’s disease patients compared to non-depressed Parkinson’s disease patients and controls.		[76]
The decreased peripheral alteration in BDNF/TrkB levels found in patients with Parkinson’s disease is directly related to dopaminergic neuron neurodegeneration.		[77]
BDNF genetic polymorphism greatly increases the risk of leucine-rich repeat kinase 2 (LRRK2) in Parkinson’s disease, particularly in subjects with older onset age.		[79]
BDNF Val66Met (rs6265, G196A) polymorphism was not associated with cognitive status in Parkinson’s disease patients, nor with Parkinson’s disease risk or onset.		[81]
The carriers of at least one BDNF 66Met allele presented a higher prevalence of cognitive impairment.		[83]
The BDNF Met allele is associated with a higher neuropsychiatric burden in Parkinson’s disease.		[84]
Carrying two copies of the BDNF rs6265 Met66 allele is associated with the reduced severity of motor symptoms and, potentially, a slower rate of progression.		[85]
The BDNF Met-allele carriers showed a significantly smaller decline in set-shifting compared with the homozygous BDNF Val-allele carriers.		[86]
The G/G genotype was significantly associated with depression and anxiety symptoms and the development of Parkinson’s disease.		[87]
The BDNF Val/Val genotype in Parkinson’s disease leads to a set of cortical and subcortical brain alterations that could promote cognitive decline.		[88]
Carriers of dopamine receptors DRD2 haplotypes and possibly the BDNF variants rs6265 and DRD3 haplotypes, were at increased risk of dyskinesia, suggesting that these genes may be involved in dyskinesia-related pathomechanisms.		[89]
Animal model		
CEBPβ (+/−) mice	Gut inflammation induces C/EBPβ activation, which leads to both BDNF and Netrin-1 reduction and triggers non-motor and motor symptoms of Parkinson’s disease.	[78]
MPTP-induced mouse model	LncRNA BDNF-AS promotes autophagy and apoptosis by ablating microRNA-125b-5p.	[90]
Cell culture		
MPP+-induced SH-SY5Y cell	BDNF-AS knockdown significantly promotes cell proliferation and suppresses apoptosis and autophagy in SH-SY5Y cells treated by MPP+. miR-125b-5p, a putative target gene of BDNF-AS, is involved in the effects of BDNF-AS on SH-SY5Y cell apoptosis and autophagy.	[90]

Additionally, the G2385R allele of the leucine-rich repeat kinase 2 (LRRK2) gene has recently been identified as a frequent genetic mutation that raises the risk of typical Parkinson’s disease exclusively among Asians [79]. BDNF genetic polymorphism may significantly increase the LRRK2-induced risk for Parkinson’s disease patients with an onset age of more than 60 years, implying a synergistic effect between the two genes [79]. In another study, the carriers of dopamine receptor DRD2 and DRD3 haplotypes, as well as the BDNF variant rs6265, were associated with an increased risk of dyskinesia, suggesting that these genes may be implicated in dyskinesia-related pathomechanisms in Parkinson’s disease patients [89]. Similar to Alzheimer’s disease, BDNF-AS has been associated with Parkinson’s disease. BDNF-AS was up-regulated in the MPTP-induced mouse model of Parkinson’s disease and dopamine neurons, as well as the MPP+-induced SH-SY5Y cell model, while miR-125b-5p was down-regulated [90]. LncRNA BDNF-AS promotes autophagy and apoptosis by ablating microRNA-125b-5p [90]. The silencing of BDNF-AS RNA can significantly increase cell proliferation and viability while inhibiting apoptosis and autophagy, suggesting that BDNF-AS might act as a potential therapeutic target for Parkinson’s disease [90].

In summary, the consistent finding of decreased serum BDNF levels in Parkinson’s disease patients support the potential use of BDNF as a biomarker for the early detection of Parkinson’s disease. There is considerable evidence suggesting is the presence of impaired BDNF signaling in the aged striatum, which may be exacerbated in people who have the BDNF rs6265. The importance of BDNF signaling in the nigrostriatal system, especially its role in maintaining synaptic function and dendritic spine density, supports the relationship between BDNF and Parkinson’s disease. However, research on the role of BDNF in Parkinson’s disease is still lacking. The precise molecular mechanisms of BDNF and its signaling cascades that lead to neurodegeneration and cognitive impairment in Parkinson’s disease remain unknown. Moreover, the contradictory results between the BDNF genotypes in the pathomechanisms of Parkinson’s disease warrant further research. Thus, there remains a great need for further exploration, especially of the targeting of modulate BDNF signaling for the treatment of aging-related neurodegenerative diseases, including Parkinson’s disease.

## 5. BDNF in Huntington’s Disease

Huntington’s disease is a neurodegenerative disorder caused by CAG repeat expansion in the huntingtin (HTT) gene. When the number of CAG repeats in the translated enlarged polyglutamine-containing HTT protein (mutant HTT [mHTT]) surpasses 36, it disrupts the normal functions of various cellular proteins, jeopardizing critical cellular machinery in neurons, astrocytes, and microglia [91]. The neuropathological changes include neuronal loss, particularly in the neocortex, striatal projection neurons, and cortico-basal ganglia-thalamocortical (CBGTC) loop, resulting in the manifestation of dysfunctional motor, cognitive, and behavioral characteristics [92,93].

The huntingtin protein promotes BDNF expression by interacting with the repressor element-1 transcription factor/neuron-restrictive silencer factor (REST/NRSF) in the cytoplasm via HAP1 and the REST-interacting LIM domain protein (RILP), preventing the translocation of this complex into the nucleus and attaching to the repressor element 1/neuron-restrictive silencer element (RE1/NRSE) found in the promoters of the BDNF and many other neuronal genes [94]. It also enhances BDNF vesicular trafficking along microtubules via a mechanism involving HAP1 and the dynactin p150 subunit [95]. Thus, mutations in the huntingtin protein default these functions, which results in a reduction in BDNF trafficking, the suppression of BDNF transcription, and a subsequent decrease in striatal BDNF.

In support of this view, reduced levels of BDNF are detected in the striata [96,97,98,99,100], brainstem regions [101], and prefrontal cortexes [102] of Huntington’s disease cell cultures, animal models, and patients (Table 3). However, inconsistent changes have been reported in the peripheral BDNF levels of Huntington’s disease patients. Huntington’s disease patients exhibited moderately increased intra-platelet BDNF levels and significantly reduced cognitive/emotional abilities, suggesting that platelet BDNF did not specifically underlie psychosocial deficits in stage II Huntington’s disease [103]. By contrast, BDNF levels were decreased in the saliva and plasma of Huntington’s disease patients compared to the control [104,105], although there was no correlation between the BDNF level and motor symptoms and cognitive impairment [105]. Furthermore, DNA methylation at the BDNF promoter IV in the blood of Huntington’s disease patients has been reported to increase [104]. Although most recent studies consistently report a decrease in BDNF levels in Huntington’s disease animal models and patients, the varying results warrant further study on the potential use of peripheral BDNF levels and BDNF-promoter methylation as biomarkers of Huntington’s disease onset and psychiatric symptoms. Several studies have investigated the probable mechanisms of the reduced BDNF levels seen in Huntington’s disease. Mutant huntingtin progressively reduced BDNF mRNA in cortical limbic, midbrain striatal afferents, motor cortex, and thalamic afferents, resulting in a gradual loss of BDNF in the subcortical and cortical striatal afferents, followed by progressive striatal neuronal loss and neurodegeneration [96]. BDNF loss in dopaminergic and limbic striatal inputs contributes to psychiatric/cognitive dysfunction, while the subsequent loss of BDNF in the thalamic and cortical motor afferents accelerates striatal degeneration, resulting in progressive involuntary movements [96]. It was also suggested that the development of Huntington’s disease is reinforced by abnormal BDNF transcription, transport, and cortical axonal secretion in the striatum, since the partial-fusion and full-fusion modes of BDNF-containing vesicles are significantly altered after the onset of Huntington’s disease symptoms [106]. There is a significant decrease in BDNF release in the cortical neurons, the BDNF levels in the striatum, and the total travel speed and length of BDNF-containing vesicles in the neurons [100]. In addition, several BDNF signaling pathways have been implicated, including mBDNF-TrkB, TrkB-Erk1/2, cAMP, MAPK, and Ras, suggesting that the downregulation of these pathways results in a decrease in BDNF expression [97,99,107].

**Table 3:** Role of BDNF in Huntington’s disease.

Observations		References
Huntington’s disease patients		
BDNF levels were significantly reduced in brainstem regions containing cardiovascular nuclei. Central administration of BDNF restored the heart rate to control levels.		[101]
In silico prediction and reporter systems prove that levels of BDNF, a central node in the miRNA-mRNA regulatory network, can be post-transcriptionally controlled by upregulated miR-10b-5p and miR-30a-5p. Reduced BDNF expression is associated with neuronal dysfunction and death in Huntington’s disease.		[102]
Huntington’s disease patients exhibited moderately increased intra-platelet BDNF levels and significantly reduced cognitive/emotional abilities. However, platelet BDNF and serotonin (5-HT) transporter (SERT) did not specifically underlie psychosocial deficits in stage-II- Huntington’s disease.		[103]
The BDNF protein levels are decreased in saliva while BDNF-promoter methylation is increased in the blood in Huntington’s disease subjects when compared to controls. Salivary BDNF measures may represent an early marker of disease onset and DNA methylation at the BDNF promoter IV could be a biomarker of psychiatric symptoms in Huntington’s disease patients.		[104]
The BDNF level was significantly lower in Huntington’s disease patients compared to the control; however, there was no correlation between the BDNF level and motor symptoms or cognitive impairment.		[105]
The pathogenesis of Huntington’s disease involved low BDNF expression, potentially mediated by the cAMP, MAPK, and Ras signaling pathways.		[107]
Animal model		
R6/2 transgenic mouse model	There is an age-dependent decrease in BDNF expression in the major sources of the afferents to the striatum. BDNF mRNA is progressively reduced in the cerebral cortexes and subcortical sources of striatal afferents, including inputs from the thalamus and the midbrain. The loss of BDNF plays an important role in motor and nonmotor abnormalities in Huntington’s disease and contributes to striatal neurodegeneration.	[96]
zQ175/zQ175|BDNF-HA/BDNF-HA mice	There is a significant decrease in mBDNF–TrkB signaling, but no induction of proBDNF-p75NTR signaling, in the striatal neurons of zQ175 mice, suggesting that the maturation of proBDNF to mBDNF remains intact. The local induction of p75NTR and sortilin is found in immature striatal oligodendrocytes and is associated with severe myelin deficits in the striata of aged zQ175 mice.	[97]
R6/2 transgenic mouse model	Striatal neurons exhibited a blunted trophic response to BDNF that was associated with the decreased activation of the TrkB-Erk1/2 signaling pathway.	[99]
zQ175 mice	There is a significant decrease in BDNF release in the cortical neurons, in the BDNF levels in the striatum, and in the total travel length and speed of BDNF-containing vesicles in the neurons.	[100]
N171-82Q mice	The BDNF levels were significantly reduced in the brainstem regions containing cardiovascular nuclei. The central administration of BDNF restored the heart rate to control levels.	[101]
Emx1-Cre/Q140 or Emx1-Cre/Q175 heterozygote mouse model	The full-fusion and partial-fusion modes of BDNF-containing vesicles were significantly altered after the onset of Huntington’s disease symptoms. The development of Huntington’s disease is reinforcedby abnormal BDNF transcription, transport, and cortical axonal secretion in the striatum.	[106]
Wild-type and age-matched symptomatic R6/2 mice	BDNF exerts neuroprotective effects on NMDA-dependent toxicity, these effects of BDNF seem specifically related to the pathological genotype, and they require endogenous A_2A_R activation.	[108]
R6/2-BDNF Huntington’s disease transgene mice	BDNF supplementation in vivo can enhance the survival and development of adult subventricular-zone-derived cells that divert to the striatum; however, augmenting BDNF levels within the olfactory bulb does not substantially improve the survival of adult-born GABAergic granule cells (GCs) in R6/2 mice at late disease stages.	[109]
3-nitropropionic acid mice model	BDNF and neurotrophin-4/5 (NT-4/5) elicit an antagonistic or synergistic effect that depends on the activation of the truncated isoform or the stimulation of the full-length isoform of the tropomyosin receptor kinase B.	[110]
Cell culture		
Huntington’s disease mutant knock-in and wild-type striatal cells	Huntington’s disease cells released lower levels of pro- and mature-BDNF. BDNF-mCherry overexpression rescued the decreased AKT phosphorylation, reduced the caspase-3 activation, and enhanced the activated ERK observed in Huntington’s disease cells.	[98]
OS-7 cell culture	BDNF and neurotrophin-4/5 (NT-4/5) elicit an antagonistic or synergistic effect that depends on the activation of the truncated isoform or the stimulation of the full-length isoform of the tropomyosin receptor kinase B.	[110]

In order to circumvent these problems, the modulation of BDNF has shown some success in alleviating symptoms in several studies in Huntington’s disease preclinical models. BDNF exerts neuroprotective effects on NMDA-dependent toxicity, and NMDA receptor-mediated excitotoxicity is hypothesized to be involved in Huntington’s disease pathogenesis [108]. These BDNF effects appear to be linked to the diseased genotype and the need for endogenous A2AR activation [108]. The levels of BDNF, a major node in the miRNA–mRNA regulation network, can be modulated post-transcriptionally by increased miR-10b-5p and miR-30a-5p, according to in silico prediction and reporter systems [102]. BDNF-mCherry overexpression reversed the caspase-3 activation, increased the AKT phosphorylation, and enhanced the activation of ERK observed in Huntington’s disease cells [98]. Furthermore, BDNF supplementation in vivo improves the survival and development of adult subventricular-zone-derived cells that divert to the striatum, although supplementing BDNF within the olfactory bulb does not significantly improve the survival of adult-born GABAergic granule cells (GCs) in R6/2 mice at late disease stages [109]. An interesting study discovered that NT-4/5 signaling occurring alone or in conjunction with BDNF-TrkB signaling might potentiate corticostriatal transmission; however, NT-4/5-TrkB-signaling can have an antagonistic effect on the BDNF-mediated modulation of corticostriatal transmission if it follows BDNF exposure in the context of striatal degeneration that mimics Huntington’s disease [110].

According to the findings described in this review, BDNF reduction in the striatum is undoubtedly linked to Huntington’s disease etiology. In animal models of Huntington’s disease, restoring cortical expression, axonal transport, and BDNF release in the striatum promotes neuronal survival and improves behavioral phenotypes. Currently, the drugs used to treat Huntington’s disease only treat its symptoms and do not halt or stop the progression of the disease. Restoring striatal BDNF levels or activating downstream signaling pathways may afford therapeutic potential in the treatment of Huntington’s disease and overcoming the functional deficits experienced by its patients. The preclinical studies on BDNF modulation offer some cause for optimism, albeit cautious, for the therapeutic potential of BDNF in Huntington’s disease.

## 6. BDNF in Amyotrophic Lateral Sclerosis (ALS)

Amyotrophic lateral sclerosis (ALS), also known as Lou Gehrig’s disease, is a fatal adult-onset neurodegenerative disease in which the upper motor neurons (UMNs, corticospinal neurons) of the motor cortex and the alpha lower motor neurons (LMNs) of the brain stem and spinal cord degenerate and die selectively [111]. These tracts control a variety of motor processes, including swallowing and breathing, which can be severely affected in ALS, with fatal consequences. With an onset typically in the fifties, the limbs gradually weaken, and mortality occurs within three years of diagnosis, primarily due to respiratory difficulties. The neuropathology associated with ALS includes astrocytosis, neuronal loss, and the deposition of aberrant phosphorylated neurofilament in the cytoplasm of neurons [112]. ALS affects 1.7 per 100,000 people each year; 90% of these cases are sporadic (sALS), while the remaining 10% exhibit familial inheritance (fALS) [113]. Mutations in the gene encoding Cu/Zn superoxide dismutase 1 (SOD1), a free radical scavenging enzyme, were the first to be identified as primary ALS mutations [114] and, since then, they have been the most studied, with extensively used mouse models of SOD1 mutations. Overall, SOD 1 mutations are responsible for 20% of fALS and 1–2% of sALS cases, with more than 180 mutations found in the SOD1 gene [115].

BDNF gene polymorphisms, specifically the G196A and C270T SNPs, are significantly associated with neurodegenerative diseases, including ALS. The frequency of the C270T T allele and the CT genotype was found to be significantly higher in the ALS group compared to controls, suggesting that BDNF C270T polymorphism could be a candidate susceptibility locus for sALS (Table 4) [116]. In addition, ALS phenotypic variability has been linked with serum BDNF levels. The BDNF serum levels were significantly lower in ALS patients expressing depressive traits and lower cognitive scores [117]. Nonetheless, no correlation was found between serum BDNF levels and disease progression speed or site of onset [117]. On another note, BDNF immunoreactivity was significantly positive in the epidermis of ALS patients, as well as moderately positive in some dermal blood vessels and glands, and these findings became more noticeable as the disease progressed [118]. These data suggest that a metabolic alteration in BDNF may occur in the skin of ALS patients.

BDNF/TrkB signaling has been demonstrated to be a key regulator of the individual and complementary actions of presynaptic activity and the subsequent muscle contraction over presynaptic kinases [119]. The disrupted connection between nervous and muscular tissues causes deficits in presynaptic activity and muscle contractility, leading to neuromuscular junction dismantling, motor neuron degeneration, and skeletal muscle denervation and atrophy [120]. Imbalances between (i) PKC isoforms and PKA subunits, (ii) BDNF and TrkB isoforms, and (iii) Munc18-1 and SNAP-25 phosphorylation ratios have been observed in symptomatic mice, while alterations in TrkB.T1 and cPKCβI have been observed in pre-symptomatic SOD1G93A mice [121]. These molecular alterations are moderately linked to the known fast-to-slow motor unit transition during the disease process, as well as the initial disease pathogenesis. Furthermore, it was shown that the deletion of BDNF receptor TrkB.T1 delays muscle weakness and spinal cord motoneuron cell death via an unknown cellular mechanism [122]. However, the deletion of TrkB.T1 did not affect the inflammatory state of the SOD1 mutant spinal cord, implying that TrkB.T1 has no effect on the activation of astrocytes or microglia [123]. Although TrkB.T1 knockout in astrocytes retains coordination and muscle strength at the early stages of the disease, the conditional deletion of TrkB.T1 in motoneurons or astrocytes does not prevent motoneuron cell death [123]. These findings imply that TrkB.T1 may be involved in ALS pathogenesis by negatively regulating the BDNF/TrkB in motor neurons. Therefore, more studies are needed to determine whether the presence of TrkB isoforms is important for planning future treatment trials with TrkB agonists in ALS.

Previous studies attempted to examine the potential value of BDNF in the treatment of ALS, although the vast majority ended in failure. Giving human neural progenitor cells engineered to express BDNF to SOD1 transgenic mice did not improve symptoms or survival [124]. Similarly, although a study reported the slowing of lung-function loss and an increase in the survival rate in ALS patients given intrathecal BDNF or recombinant methionyl BDNF (rhmetBDNF) [125], most other studies reported no or very little improvement [125,126,127,128]. More recently, other approaches to modulating BDNF for the treatment of ALS have been attempted. The non-toxic C-terminal fragment of the tetanus toxin (TTC) heavy chain has been explored as a neuroprotective agent and a carrier molecule to the CNS. Treatment with TTC alone and with the fusion molecule BDNF-TTC significantly delayed the onset of functional deficits and symptoms in SOD1G93A mice [129]. The treatment partially preserved muscle innervation, increased the number of surviving motoneurons in the L2 spinal cord segment, inhibited pro-apoptotic protein targets (caspase-3 and Bax), and phosphorylated Akt and ERK in the spinal cord [129]. Similarly, exogenous BDNF supplementation reversed autocrine expression and organellar ultrastructural changes, inhibited apoptosis, and completely revived choline acetyltransferase (ChAT) expression; however, it may not be completely receptor-mediated, as the TrkB levels were not restored [130]. The incomplete revival at the ultrastructural level indicates that elements other than BDNF are required for the near-total protection of motor neurons, which also helps to explain why clinical studies using BDNF in ALS patients have only had limited success. Furthermore, it was discovered that transplanting BDNF-overexpressing hUC-MSC-derived motor neurons into hSOD1G93A mice can improve motor performance and extend longevity [131]. This study suggests the combination of BDNF with stem-cell-derived motor neurons as a new therapeutic strategy for ALS.

**Table 4:** Role of BDNF in amyotrophic lateral sclerosis.

Observations		References
ALS patients		
The frequency of the CT genotype and the C270T T allele was significantly higher in the ALS group than in the controls. BDNF C270T polymorphism may be a candidate susceptibility locus for sALS, at least in Han Chinese populations.		[116]
The BDNF serum levels did not differ between the patients and the controls, although ∼25% lower levels characterized the patients carrying a depressive trait. The BDNF serum levels were significantly lower in the ALS patients expressing lower cognitive scores.		[117]
The BDNF immunoreactivity was markedly positive in the epidermis and moderately positive in some dermal blood vessels and glands. A metabolic BDNF alteration may take place in the skin of ALS patients.		[118]
Animal model		
Pre- and symptomatic SOD1G93A mice	There are imbalances between (I) BDNF and TrkB isoforms, (II) PKC isoforms and PKA subunits, and (III) Munc18-1 and SNAP-25 phosphorylation ratios in symptomatic mice. Changes in TrkB.T1 and cPKCβI are frequently observed in pre-symptomatic mice.	[121]
SOD1G93A T1-/- ALS mouse model	TrkB.T1 deletion significantly delayed the onset of motor-neuron degeneration and the development of muscle weakness.	[122]
ALS G93A SOD1 animal model	TrkB.T1 may limit BDNF signaling to motoneurons via a non-cellular autonomous mechanism.	[123]
SOD1G93A transgenic mice	Significant improvements in behavioral and electrophysiological results, motoneuron survival, and anti-apoptotic/survival-activated pathways were observed with BDNF-TTC treatment. However, no synergistic effect was found for this fusion molecule.	[129]
hSOD1G93A mice	The transplantation of BDNF-overexpressing hUC-MSC-derived motor neurons improves motor performance and prolongs the survival of hSOD1G93A mice.	[131]
Cell culture		
NSC-34 cells	The exogenous BDNF supplementation ameliorated most, but not all, degenerative changes. BDNF supplementation reversed autocrine expression; however, it may not be completely receptor-mediated, as the TrkB levels were not restored. BDNF completely revived ChAT expression, inhibited apoptosis, and partially reversed organellar ultrastructural changes.	[130]

Riluzole, a glutamatergic neurotransmission inhibitor, and Edaravone, an antioxidant medication, are the only two FDA-approved treatments for ALS to date. They are found to have modest benefits for survival and are effective at halting ALS progression during its early stages [132]. However, a few dose-related side effects of their usage have been reported, such as nausea, asthenia, and elevated liver-enzyme levels [132]. Considering the importance of BDNF and its signaling pathways in ALS disease onset and progression, its therapeutic potential should be exploited and further explored. Although earlier studies using intrathecal BDNF failed to produce positive outcomes, these findings do not rule out the possibility of BDNF’s use in ALS. Issues may arise from the intrathecal BDNF protein delivery, including its passage across the cerebrospinal fluid (CSF)–brain barrier, as well as the protein’s low distribution rate and short half-life, which could explain the poor clinical outcomes. The presence of abundant truncated TrkB receptors in the brain can mop up and impede protein delivery, causing insufficient levels of BDNF to reach the corticospinal neurons. Furthermore, BDNF also binds to the p75NTR receptor, which has been linked to the neuropathology of ALS, whereas a p75NTR antagonist has been demonstrated to decrease ALS progression in SOD1 mice [133]. In addition, combination therapies or fusion molecules, such as BDNF-TTC, may work best for treating ALS. Moreover, the use of a basket design when conducting clinical trials may be more effective for ALS. Overall, these difficulties must be addressed to offer BDNF-associated treatment a fair chance as a possible therapy for this crippling disease.

## 7. BDNF in Other Neurodegenerative Diseases

Spinocerebellar ataxia type 1 (SCA1) is a fatal neurodegenerative disease caused by the aberrant amplification of CAG repeats in the Ataxin1 (ATXN1) gene. SCA1 is characterized by cerebellar neurodegeneration, motor deficits, and changes in gliosis and gene expression. Extrinsic BDNF delivery has been shown to delay the onset of motor deficits and Purkinje neuron pathology in ATXN1(82Q) mice, implying that it could be used as a new SCA1 treatment [134]. Another study reported that the post-symptomatic delivery of extrinsic BDNF alleviated cerebellar pathologies and motor deficits, such as astrogliosis and the dendritic atrophy of Purkinje cells, although the expression of the Purkinje cell gene was not altered [135]. In spinocerebellar ataxia type 6 (SCA6), an autosomal-dominant neurodegenerative disease caused by a small expansion of CAG repeat encoding polyglutamine (polyQ) in the gene for α1A voltage-dependent calcium channel (Cav2.1) and characterized by Purkinje cell neuronal loss, the BDNF mRNA levels and BDNF protein expression in the SCA6 cerebellum significantly reduced, whilst numerous BDNF-immunoreactive granules were found in the dendrites of SCA6 Purkinje cells [136]. This suggests that the SCA6 pathogenic mechanism is associated with the abnormal localization of BDNF proteins and a reduction in BDNF mRNA expression. A comprehensive transcriptome analysis in Friedreich’s ataxia (FRDA) patients, a hereditary neurodegenerative disease characterized by guanine-adenine-adenine (GAA) nucleotide repeat expansion in the first intron of the frataxin (FXN) gene, identified BDNF and FXN as novel targets of miRNAs [137].

In multiple sclerosis (MS) patients, the serum levels of BDNF have the potential to be used as severity biomarkers [138,139,140]. The percentage of BDNF gene methylation may be used as a predictive marker for the progression of the disease toward severe disability in MS patients [141]. In addition, BDNF Val66Met polymorphism (rs6265) has been shown to modulate neurodegeneration and inflammation in the early phases of MS [142]. Val66Met MS patients had a higher volume of hippocampal subfields than BDNF Val66Val MS patients, suggesting that BNDF Val66Met polymorphism may protect MS patients from cognitive impairment and hippocampal atrophy, and that the BDNF genotype may be a biomarker for predicting cognitive prognosis [143]. BDNF Val66Met polymorphism has also been shown to protect against cognitive impairment and improve motor recovery in MS patients [144,145].

Additionally, BDNF has been linked to the syndromes associated with neurodegenerative diseases such as dementia and MCI. Serum BDNF levels are associated with the risk and severity of Alzheimer’s disease dementia [146,147], whereas BDNF Val66Met polymorphisms are associated with the phenotypic variability seen in patients with frontotemporal lobar degeneration (FTLD) syndromes [148]. Decreased hippocampal BDNF expression, with significant neuronal damage and cognitive impairment, was observed in a vascular dementia rat model [149]. The cognitive impairment in vascular dementia involved the BDNF-ERK-CREB pathway [150]. Additionally, BDNF Val66Met polymorphisms are associated with reduced BDNF serum levels in patients with MCI [151] and with poor cognitive functions in patients with aMCI [152]. The potential use of the peripheral BDNF level as a biomarker for MCI has also been discussed [153,154,155].

## 8. Conclusions

In this review, we discussed the role of BDNF and the underlying molecular mechanism in the pathophysiology of neurodegenerative diseases. BDNF regulates a wide range of processes in the brain, sometimes with contrasting effects. This can be explained by its unique synthesis pattern, which includes several biologically active isoforms that interact with various types of receptors, ultimately initiating multiple signaling pathways. BDNF Val66Met polymorphism influences the risk of neurodegenerative disease development, onset, and pathomechanisms. Because of the importance of BDNF signaling in the nigrostriatal system, especially for synaptic function and maintaining dendritic spine density, impaired BDNF signaling in association with rs6265 SNV and/or aging is likely to have a significant and negative impact on basal ganglia plasticity and function in health and disease.

The evidence discussed in this article indicates that deficiency in BDNF and TrkB signaling may play a role in the pathophysiology of Alzheimer’s disease, Parkinson’s disease, and Huntington’s disease. Consequently, this suggests that peripheral BDNF or TrkB levels and other associated factors, such as the role of TrkB isoforms, BDNF promoter methylation, BDNF-AS, and the BDNF pro-to-mature-domain ratio, may be used as biomarkers for early disease detection, risk, mnemonic symptoms, and conversion. The modulation of these factors may offer neuroprotective effects and, potentially, treatment for some neurodegenerative diseases. The primary studies on BDNF therapy in patients and animal models provide compelling evidence that supplying exogenous BDNF or increasing endogenous BDNF production could have therapeutic effects. Nevertheless, the application of BDNF therapy needs to be strategized for it to be efficacious, safe, and administered in an appropriate amount and spatiotemporal context. Furthermore, other interventions, such as the chronic administration of fluoxetine, exercise, and environmental enrichment have been shown to enhance serum BDNF levels, cognitive performance, and neurogenesis, suggesting the potential of BDNF modulation for the treatment of neurodegenerative diseases. However, various methodological and safety issues for patients need to be considered before this strategy can be extensively utilized. In addition, future research should concentrate on determining the multifunctional roles of BDNF in different brain regions, as well as closely controlled clinical trials.

## Data Availability

Not applicable.

## Abbreviations

Amnestic mild cognitive impairment: aMCI. Amyloid β: Aβ. Amyotrophic lateral sclerosis: ALS. Ataxin 1: ATXN1. BDNF antisense: BDNF-AS. Brain-derived neurotrophic factor: BDNF. c-Jun amino terminal kinase: JNK. Calcium-calmodulin-dependent protein kinase: CAMK. cAMP response element-binding protein: CREB. Cerebral metabolic rate for glucose: CMRgl. Cerebrospinal fluid: CSF. Choline acetyltransferase: ChAT. Cortico-basal ganglia-thalamocortical: CBGTC. Dopamine: DA. Extracellular-signal-regulated kinase ½: ERK 1/2. Familial amyotrophic lateral sclerosis: fALS. Frataxin: FXN. Friedreich’s ataxia: FRDA. Frontotemporal lobar degeneration: FTLD. Gamma-amino butyric acid: GABA. Guanine-adenine-adenine: GAA. Guanosine triphosphate hydrolases: GTP. Huntingtin: HTT. Janus kinase: JAK. Late-phase long-term potentiation: L-LTP. Leucine-rich repeat kinase 2: LRRK2. Long-term depression: LTD. Lower motor neurons: LMNs. Mitogen-activated protein kinase: MAPK. Multiple sclerosis: MS. Mutant HTT: mHTT. N-methyl-D-aspartate receptor: NMDAR. Nerve growth factor: NGF. Neural stem cells: NSCs. Neurotrophin-3: NT3. Neurotrophin-4/5: NT4/5. Neurotrophin receptor-interacting factor: NRIF. Non-toxic C-terminal fragment of the tetanus toxin: TTC. Nuclear factor kappa B: NF-kB. p75 pan-neurotrophin receptor: p75NTR; Pars compacta of the substantia nigra: SNpc; Phosphatase and tension homolog: PTEN. Phosphoinositide 3-kinases-protein kinase B: PI3K/AKT. Phospholipase C: PLC. Polyglutamine: polyQ. Protein kinase C: PKC. Recombinant methionyl BDNF: rhmetBDNF. Repressor element 1/neuron-restrictive silencer element: RE1/NRSE. Repressor element-1 transcription factor/neuron-restrictive silencer factor: REST/NRSF. REST-interacting LIM domain protein: RILP. Restless legs syndrome: RLS. RhoA/Rho-associated kinase: ROCK. Spinocerebellar ataxia type 1: SCA1. Spinocerebellar ataxia type 6: SCA6. Sporadic amyotrophic lateral sclerosis: sALS. Striatal medium spiny neurons: MSNs. Substantia nigra: SN. Superoxide dismutase 1: SOD1. Tumor necrosis factor receptor-associated factor 6: TRAF6. Tyrosine receptor kinase B: TrkB. Upper motor neurons: UMNs. Vacuolar protein-sorting 10 protein: Vps10p.

## References

[1] Hou Y., Dan X., Babbar M., Wei Y., Hasselbalch S.G., Croteau D.L., Bohr V.A. (2019). Ageing as a Risk Factor for Neurodegen-erative Disease. Nat. Rev. Neurol..

[2] Dechant G., Neumann H. (2003). Neurotrophins. Mol. Cell. Biol. Neuroprotection CNS.

[3] Foltran R.B., Diaz S.L. (2016). BDNF Isoforms: A Round Trip Ticket between Neurogenesis and Serotonin?. J. Neurochem..

[4] Mowla S.J., Farhadi H.F., Pareek S., Atwal J.K., Morris S.J., Seidah N.G., Murphy R.A. (2001). Biosynthesis and Post-translational Processing of the Precursor to Brain-derived Neurotrophic Factor. J. Biol. Chem..

[5] Je H.S., Yang F., Ji Y., Nagappan G., Hempstead B.L., Lu B. (2012). Role of pro-brain-derived neurotrophic factor (proBDNF) to mature BDNF conversion in activity-dependent competition at developing neuromuscular synapses. Proc. Natl. Acad. Sci. USA.

[6] Vafadari B., Salamian A., Kaczmarek L. (2016). MMP-9 in Translation: From Molecule to Brain Physiology, Pathology, and Therapy. J. Neurochem..

[7] Dieni S., Matsumoto T., Dekkers M., Rauskolb S., Ionescu M.S., Deogracias R., Gundelfinger E.D., Kojima M., Nestel S., Frotscher M. (2012). BDNF and its pro-peptide are stored in presynaptic dense core vesicles in brain neurons. J. Cell Biol..

[8] Yang J.-L., Lin Y.-T., Chuang P.-C., Bohr V.A., Mattson M.P. (2013). BDNF and Exercise Enhance Neuronal DNA Repair by Stimulating CREB-Mediated Production of Apurinic/Apyrimidinic Endonuclease 1. Neuromolecular Med..

[9] Deinhardt K., Chao M.V. (2014). Shaping Neurons: Long and Short Range Effects of Mature and ProBDNF Signalling upon Neuronal Structure. Neuropharmacology.

[10] Nykjaer A., Willnow T.E. (2012). Sortilin: A receptor to regulate neuronal viability and function. Trends Neurosci..

[11] Simmons D.A. (2017). Modulating Neurotrophin Receptor Signaling as a Therapeutic Strategy for Huntington’s Disease. J. Huntington’s Dis..

[12] Song W., Volosin M., Cragnolini A.B., Hempstead B.L., Friedman W.J. (2010). ProNGF Induces PTEN via p75NTR to Suppress Trk-Mediated Survival Signaling in Brain Neurons. J. Neurosci..

[13] Sandhya V.K., Raju R., Verma R., Advani J., Sharma R., Radhakrishnan A., Nanjappa V., Narayana J., Somani B.L., Mukherjee K.K. (2013). A network map of BDNF/TRKB and BDNF/p75NTR signaling system. J. Cell Commun. Signal..

[14] Colucci-D’Amato L., Speranza L., Volpicelli F. (2020). Neurotrophic Factor BDNF, Physiological Functions and Therapeutic Potential in Depression, Neurodegeneration and Brain Cancer. Int. J. Mol. Sci..

[15] Minichiello L. (2009). TrkB signalling pathways in LTP and learning. Nat. Rev. Neurosci..

[16] Gonzalez A., Moya-Alvarado G., Gonzalez-Billaut C., Bronfman F.C. (2016). Cellular and molecular mechanisms regulating neuronal growth by brain-derived neurotrophic factor. Cytoskeleton.

[17] Jaworski J., Spangler S., Seeburg D.P., Hoogenraad C.C., Sheng M. (2005). Control of Dendritic Arborization by the Phospho-inositide-3′-Kinase–Akt–Mammalian Target of Rapamycin Pathway. J. Neurosci..

[18] Panja D., Kenney J.W., D’Andrea L., Zalfa F., Vedeler A., Wibrand K., Fukunaga R., Bagni C., Proud C.G., Bramham C.R. (2014). Two-Stage Translational Control of Dentate Gyrus LTP Consolidation Is Mediated by Sustained BDNF-TrkB Signaling to MNK. Cell Rep..

[19] Zhao H., Alam A., San C.-Y., Eguchi S., Chen Q., Lian Q., Ma D. (2017). Molecular mechanisms of brain-derived neurotrophic factor in neuro-protection: Recent developments. Brain Res..

[20] Lin G., Bella A.J., Lue T.F., Lin C. (2006). Brain-Derived Neurotrophic Factor (BDNF) Acts Primarily via the JAK/STAT Pathway to Promote Neurite Growth in the Major Pelvic Ganglion of the Rat: Part 2. J. Sex. Med..

[21] Sakuragi S., Tominaga-Yoshino K., Ogura A. (2013). Involvement of TrkB- and p75NTR-signaling pathways in two contrasting forms of long-lasting synaptic plasticity. Sci. Rep..

[22] World Health Organization WHO Reveals Leading Causes of Death and Disability Worldwide: 2000–2019. https://www.who.int/news/item/09-12-2020-who-reveals-leading-causes-of-death-and-disability-worldwide-2000-2019.

[23] (2019). Alzheimer’s Association 2019 Alzheimer’s Disease Facts and Figures. Alzheimer’s Dement..

[24] Selkoe D.J., Hardy J. (2016). The amyloid hypothesis of Alzheimer’s disease at 25 years. EMBO Mol. Med..

[25] Blennow K., Zetterberg H. (2022). The Neurochemistry of Alzheimer’s Disease: One of the Most Common Causes of Reduced Ca-pability in the Adult Population. A Multidisciplinary Approach to Capability in Age and Ageing.

[26] Serrano-Pozo A., Frosch M.P., Masliah E., Hyman B.T. (2011). Neuropathological Alterations in Alzheimer Disease. Cold Spring Harb. Perspect. Med..

[27] Holsinger R., Schnarr J., Henry P., Castelo V.T., Fahnestock M. (2000). Quantitation of BDNF mRNA in human parietal cortex by competitive reverse transcription-polymerase chain reaction: Decreased levels in Alzheimer’s disease. Mol. Brain Res..

[28] Connor B., Young D., Yan Q., Faull R., Synek B., Dragunow M. (1997). Brain-derived neurotrophic factor is reduced in Alzheimer’s disease. Mol. Brain Res..

[29] Garzon D., Yu G., Fahnestock M. (2002). A New Brain-derived Neurotrophic Factor Transcript and Decrease Inbrain-derived Neurotrophic Factor Transcripts 1, 2 and 3 in Alzheimer’s Disease Parietal Cortex. J. Neurochem..

[30] Hock C., Heese K., Hulette C., Rosenberg C., Otten U. (2000). Region-Specific Neurotrophin Imbalances in Alzheimer Disease: Decreased Levels of Brain-Derived Neurotrophic Factor and Increased Levels of Nerve Growth Factor in Hippocampus and Cortical Areas. Arch. Neurol..

[31] Laske C., Stransky E., Leyhe T., Eschweiler G.W., Maetzler W., Wittorf A., Soekadar S., Richartz E., Koehler N., Bartels M. (2007). BDNF Serum and CSF Concentrations in Alzheimer’s Disease, Normal Pressure Hydrocephalus and Healthy Controls. J. Psychiatr. Res..

[32] Gezen-Ak D., Dursun E., Hanağası H., Bilgiç B., Lohman E., Araz S., Atasoy I.L., Alaylıoğlu M., Önal B., Gürvit H. (2013). BDNF, TNFα, HSP90, CFH, and IL-10 Serum Levels in Patients with Early or Late Onset Alzheimer’s Disease or Mild Cognitive Impairment. J. Alzheimer’s Dis..

[33] Pláteník J., Fišar Z., Buchal R., Jirák R., Kitzlerova E., Zverova M., Raboch J. (2014). GSK3β, CREB, and BDNF in peripheral blood of patients with Alzheimer’s disease and depression. Prog. Neuro-Psychopharmacol. Biol. Psychiatry.

[34] Faria M.C., Gonçalves G.S., Rocha N.P., Moraes E.N., Bicalho M.A., Gualberto Cintra M.T., Jardim de Paula J., José Ravic de Miranda L.F., Clayton de Souza Ferreira A., Teixeira A.Ô.L. (2014). Increased plasma levels of BDNF and inflammatory markers in Alzheimer’s disease. J. Psychiatr. Res..

[35] Kim H.W.S.H. (2015). Differences in BDNF Serum Levels in Patients with Alzheimer’s Disease and Mild Cognitive Impairment. Afr. J. Psychiatry.

[36] Qin X.-Y., Cao C., Cawley N.X., Liu T.-T., Yuan J., Loh Y.P., Cheng Y. (2016). Decreased peripheral brain-derived neurotrophic factor levels in Alzheimer’s disease: A meta-analysis study (N=7277). Mol. Psychiatry.

[37] Ng T.K.S., Ho C.S.H., Tam W.W.S., Kua E.H., Ho R.C.-M. (2019). Decreased Serum Brain-Derived Neurotrophic Factor (BDNF) Levels in Patients with Alzheimer’s Disease (AD): A Systematic Review and Meta-Analysis. Int. J. Mol. Sci..

[38] Du Y., Wu H.-T., Qin X.-Y., Cao C., Liu Y., Cao Z.-Z., Cheng Y. (2018). Postmortem Brain, Cerebrospinal Fluid, and Blood Neu-rotrophic Factor Levels in Alzheimer’s Disease: A Systematic Review and Meta-Analysis. J. Mol. Neurosci..

[39] Mori Y., Tsuji M., Oguchi T., Kasuga K., Kimura A., Futamura A., Sugimoto A., Kasai H., Kuroda T., Yano S. (2021). Serum BDNF as a Potential Biomarker of Alzheimer’s Disease: Verification Through Assessment of Serum, Cerebrospinal Fluid, and Medial Temporal Lobe Atrophy. Front. Neurol..

[40] Wang Z.-H., Xiang J., Liu X., Yu S.P., Manfredsson F.P., Sandoval I.M., Wu S., Wang J.-Z., Ye K. (2019). Deficiency in BDNF/TrkB Neurotrophic Activity Stimulates δ-Secretase by Upregulating C/EBPβ in Alzheimer’s Disease. Cell Rep..

[41] Rosa E., Mahendram S., Ke Y.D., Ittner L.M., Ginsberg S.D., Fahnestock M. (2016). Tau downregulates BDNF expression in animal and cellular models of Alzheimer’s disease. Neurobiol. Aging.

[42] Sen A., Nelson T.J., Alkon D.L., Hongpaisan J. (2018). Loss in PKC Epsilon Causes Downregulation of MnSOD and BDNF Expression in Neurons of Alzheimer’s Disease Hippocampus. J. Alzheimer’s Dis..

[43] Jiao S.-S., Shen L.-L., Zhu C., Bu X.-L., Liu Y.-H., Liu C.-H., Yao X.-Q., Zhang L.-L., Zhou H.-D., Walker D.G. (2016). Brain-derived neurotrophic factor protects against tau-related neurodegeneration of Alzheimer’s disease. Transl. Psychiatry.

[44] Wu C.-C., Lien C.-C., Hou W.-H., Chiang P.-M., Tsai K.-J. (2016). Gain of BDNF Function in Engrafted Neural Stem Cells Promotes the Therapeutic Potential for Alzheimer’s Disease. Sci. Rep..

[45] De Pins B., Cifuentes-Díaz C., Farah A.T., López-Molina L., Montalban E., Sancho-Balsells A., López A., Ginés S., Delgado-García J.M., Alberch J. (2019). Conditional BDNF delivery from astrocytes rescues memory deficits, spine density and synaptic properties in the 5xFAD mouse model of Alzheimer disease. J. Neurosci..

[46] Chang L., Wang Y., Danjie J., Dai D., Xu X., Jiang D., Zhongming C., Ye H., Zhang X., Zhou X. (2014). Elevation of Peripheral BDNF Promoter Methylation Links to the Risk of Alzheimer’s Disease. PLoS ONE.

[47] Li G.-D., Bi R., Zhang D.-F., Xu M., Luo R., Wang D., Fang Y., Li T., Zhang C., Yao Y.-G. (2017). Female-specific effect of the BDNF gene on Alzheimer’s disease. Neurobiol. Aging.

[48] Liu Y.-H., Jiao S.-S., Wang Y.-R., Bu X.-L., Yao X.-Q., Xiang Y., Wang Q.-H., Wang L., Deng J., Li J. (2014). Associations between ApoEε4 Carrier Status and Serum BDNF Levels—New Insights into the Molecular Mechanism of ApoEε4 Actions in Alzheimer’s Disease. Mol. Neurobiol..

[49] Lin P.-H., Tsai S.-J., Huang C.-W., Mu-En L., Hsu S.-W., Lee C.-C., Chen N.-C., Chang Y.-T., Lan M.-Y., Chang C.-C. (2016). Dose-dependent genotype effects of BDNF Val66Met polymorphism on default mode network in early stage Alzheimer’s disease. Oncotarget.

[50] Borroni B., Grassi M., Archetti S., Costanzi C., Bianchi M., Caimi L., Caltagirone C., Di Luca M., Padovani A. (2015). BDNF Genetic Variations Increase the Risk of Alzheimer’s Disease-Related Depression. Handbook of Depression in Alzheimer’s Disease.

[51] Aarons T., Bradburn S., Robinson A., Payton A., Pendleton N., Murgatroyd C. (2019). Dysregulation of BDNF in Prefrontal Cortex in Alzheimer’s Disease. J. Alzheimer’s Dis..

[52] Azizi-Aghaali R., Khalaj-Kondori M., Zeinalzadeh N., Hoseinpour Feizi M.A., Farhoudi M., Talebi M. (2018). Comparison be-tween the Plasma Levels of Long Noncoding RNA BDNF-as in Patients with Alzheimer’s Disease and Healthy Subjects. J. Babol Univ. Med. Sci..

[53] Ding Y., Luan W., Shen X., Wang Z., Cao Y. (2021). LncRNA BDNF-AS as ceRNA regulates the miR-9-5p/BACE1 pathway affecting neurotoxicity in Alzheimer’s disease. Arch. Gerontol. Geriatr..

[54] Bharani K.L., Ledreux A., Gilmore A., Carroll S.L., Granholm A.-C. (2019). Serum pro-BDNF levels correlate with phospho-tau staining in Alzheimer’s disease. Neurobiol. Aging.

[55] Xie B., Liu Z., Liu W., Jiang L., Zhang R., Cui D., Zhang Q., Xu S. (2017). DNA Methylation and Tag SNPs of the BDNF Gene in Conversion of Amnestic Mild Cognitive Impairment into Alzheimer’s Disease: A Cross-Sectional Cohort Study. J. Alzheimer’s Dis..

[56] Xie B., Xu Y., Liu Z., Liu W., Jiang L., Zhang R., Cui D., Zhang Q., Xu S. (2017). Elevation of Peripheral BDNF Promoter Methylation Predicts Conversion from Amnestic Mild Cognitive Impairment to Alzheimer’s Disease: A 5-Year Longitudinal Study. J. Alzheimer’s Dis..

[57] Bessi V., Mazzeo S., Bagnoli S., Padiglioni S., Carraro M., Piaceri I., Bracco L., Sorbi S., Nacmias B. (2020). The implication of BDNF Val66Met polymorphism in progression from subjective cognitive decline to mild cognitive impairment and Alzheimer’s disease: A 9-year follow-up study. Eur. Arch. Psychiatry Clin. Neurosci..

[58] Stonnington C.M., Velgos S.N., Chen Y., Syed S., Huentelman M., Thiyyagura P., Lee W., Richholt R., Caselli R.J., Locke D.E. (2020). Interaction Between BDNF Val66Met and APOE4 on Biomarkers of Alzheimer’s Disease and Cognitive Decline. J. Alzheimer’s Dis..

[59] Rantamäki T., Kemppainen S., Autio H., Stavén S., Koivisto H., Kojima M., Antila H., Miettinen P.O., Kärkkäinen E., Karpova N. (2013). The Impact of Bdnf Gene Deficiency to the Memory Impairment and Brain Pathology of APPswe/PS1dE9 Mouse Model of Alzheimer’s Disease. PLoS ONE.

[60] Lim J.Y., Reighard C.P., Crowther D.C. (2015). The pro-domains of neurotrophins, including BDNF, are linked to Alzheimer’s disease through a toxic synergy with Aβ. Hum. Mol. Genet..

[61] Guo C.-C., Jiao C.-H., Gao Z.-M. (2018). Silencing of LncRNA BDNF-AS attenuates Aβ_25-35_-induced neurotoxicity in PC12 cells by suppressing cell apoptosis and oxidative stress. Neurol. Res..

[62] Tysnes O.-B., Storstein A. (2017). Epidemiology of Parkinson’s Disease. J. Neural Transm..

[63] Yang W., Hamilton J.L., Kopil C., Beck J.C., Tanner C.M., Albin R.L., Dorsey E.R., Dahodwala N., Cintina I., Hogan P. (2020). Current and Projected Future Economic Burden of Parkinson’s Disease in the U.S. NPJ Park. Dis..

[64] Doherty K.M., van de Warrenburg B.P., Peralta M.C., Moriyama L.S., Azulay J.-P., Gershanik O.S., Bloem B.R. (2011). Postural deformities in Parkinson’s disease. Lancet Neurol..

[65] Altar C.A., Boylan C.B., Jackson C., Hershenson S., Miller J., Wiegand S.J., Lindsay R.M., Hyman C. (1992). Brain-derived neurotrophic factor augments rotational behavior and nigrostriatal dopamine turnover in vivo. Proc. Natl. Acad. Sci. USA.

[66] Hyman C., Hofer M., Barde Y., Juhasz M., Yancopoulos G.D., Squinto S.P., Lindsay R.M. (1991). BDNF is a neurotrophic factor for dopaminergic neurons of the substantia nigra. Nature.

[67] Sauer H., Fischer W., Nikkhah G., Wiegand S.J., Brundin P., Lindsay R.M., Björklund A. (1993). Brain-derived neurotrophic factor enhances function rather than survival of intrastriatal dopamine cell-rich grafts. Brain Res..

[68] Cunha C., Brambilla R., Thomas K.L. (2010). A Simple Role for BDNF in Learning and Memory?. Front. Mol. Neurosci..

[69] Lai K.-O., Ip N.Y. (2013). Structural Plasticity of Dendritic Spines: The Underlying Mechanisms and Its Dysregulation in Brain Dis-orders. Biochim. Biophys. Acta (BBA)-Mol. Basis Dis..

[70] Collier T.J., Sortwell C.E. (1999). Therapeutic potential of nerve growth factors in Parkinson’s disease. Drugs Aging.

[71] Khalil H., Alomari M., Khabour O.F., Al-Hieshan A., Bajwa J.A. (2016). Relationship of circulatory BDNF with cognitive deficits in people with Parkinson’s disease. J. Neurol. Sci..

[72] Wang Y., Liu H., Du X.-D., Zhang Y., Yin G., Zhang B.-S., Soares J.C., Zhang X.Y. (2017). Association of low serum BDNF with depression in patients with Parkinson’s disease. Park. Relat. Disord..

[73] Hernández-Vara J., Sáez-Francàs N., Lorenzo-Bosquet C., Corominas-Roso M., Cuberas-Borròs G., Pozo S.L.-D., Carter S., Armengol-Bellapart M., Castell-Conesa J. (2020). BDNF levels and nigrostriatal degeneration in “drug naïve” Parkinson’s disease patients. An “in vivo” study using I-123-FP-CIT SPECT. Park. Relat. Disord..

[74] Wang Y., Liu H., Zhang B.-S., Soares J.C., Zhang X.Y. (2016). Low BDNF is associated with cognitive impairments in patients with Parkinson’s disease. Park. Relat. Disord..

[75] Huang Y.-X., Zhang Q.-L., Huang C.-L., Wu W.-Q., Sun J.-W. (2021). Association of Decreased Serum BDNF With Restless Legs Syndrome in Parkinson’s Disease Patients. Front. Neurol..

[76] Huang Y., Huang C., Zhang Q., Wu W., Sun J. (2020). Serum BDNF discriminates Parkinson’s disease patients with depression from without depression and reflect motor severity and gender differences. J. Neurol..

[77] Huang Y., Huang C., Yun W. (2019). Peripheral BDNF/TrkB protein expression is decreased in Parkinson’s disease but not in Essential tremor. J. Clin. Neurosci..

[78] Ahn E.H., Kang S.S., Liu X., Cao X., Choi S.Y., Musazzi L., Mehlen P., Ye K. (2020). BDNF and Netrin-1 repression by C/EBPβ in the gut triggers Parkinson’s disease pathologies, associated with constipation and motor dysfunctions. Prog. Neurobiol..

[79] Liu J., Zhou Y., Wang C., Wang T., Zheng Z., Chan P. (2012). Brain-derived neurotrophic factor (BDNF) genetic polymorphism greatly increases risk of leucine-rich repeat kinase 2 (LRRK2) for Parkinson’s disease. Park. Relat. Disord..

[80] Karamohamed S., Latourelle J.C., Racette B.A., Perlmutter J.S., Wooten G.F., Lew M., Klein C., Shill H., Golbe L.I., Mark M.H. (2005). BDNF genetic variants are associated with onset age of familial Parkinson disease: GenePD Study. Neurology.

[81] Białecka M., Kurzawski M., Roszmann A., Robowski P., Sitek E.J., Honczarenko K., Mak M., Deptuła-Jarosz M., Gołąb-Janowska M., Droździk M. (2014). BDNF G196A (Val66Met) polymorphism associated with cognitive impairment in Parkinson’s disease. Neurosci. Lett..

[82] Chen Z.-Y., Patel P.D., Sant G., Meng C.-X., Teng K.K., Hempstead B.L., Lee F.S. (2004). Variant Brain-Derived Neurotrophic Factor (BDNF)(Met66) Alters the Intracellular Trafficking and Activity-Dependent Secretion of Wild-Type BDNF in Neuro-secretory Cells and Cortical Neurons. J. Neurosci..

[83] Altmann V., Schumacher-Schuh A.F., Rieck M., Callegari-Jacques S.M., Rieder C.R., Hutz M.H. (2016). Val66Met BDNF polymorphism is associated with Parkinson’s disease cognitive impairment. Neurosci. Lett..

[84] Ramezani M., Ruskey J.A., Martens K., Kibreab M., Javer Z., Kathol I., Hammer T., Cheetham J., Leveille E., Martino D. (2021). Association Between BDNF Val66Met Polymorphism and Mild Behavioral Impairment in Patients with Parkinson’s Disease. Front. Neurol..

[85] Fischer D.L., Auinger P., Goudreau J.L., Paumier K.L., Cole-Strauss A., Kemp C.J., Lipton J.W., Sortwell C.E. (2018). Bdnf variant is associated with milder motor symptom severity in early-stage Parkinson’s disease. Park. Relat. Disord..

[86] van der Kolk N.M., Speelman A.D., van Nimwegen M., Kessels R.P., IntHout J., Hakobjan M., Munneke M., Bloem B.R., van de Warrenburg B.P. (2015). BDNF polymorphism associates with decline in set shifting in Parkinson’s disease. Neurobiol. Aging.

[87] Cagni F.C., Campêlo C.L.D.C., Coimbra D.G., Barbosa M.R., Júnior L.G.O., Neto A.B.S., Ribeiro A.M., Júnior C.D.O.G., de Andrade T., Silva R.H. (2017). Association of BDNF Val66MET Polymorphism with Parkinson’s Disease and Depression and Anxiety Symptoms. J. Neuropsychiatry Clin. Neurosci..

[88] Sampedro F., Marín-Lahoz J., Martínez-Horta S., Pagonabarraga J., Kulisevsky J. (2019). Pattern of cortical thinning associated with the BDNF Val66Met polymorphism in Parkinson’s disease. Behav. Brain Res..

[89] Kusters C.D., Paul K.C., Guella I., Bronstein J.M., Sinsheimer J.S., Farrer M.J., Ritz B.R. (2017). Dopamine receptors and BDNF -haplotypes predict dyskinesia in Parkinson’s disease. Park. Relat. Disord..

[90] Fan Y., Zhao X., Lu K., Cheng G. (2020). LncRNA BDNF-AS promotes autophagy and apoptosis in MPTP-induced Parkinson’s disease via ablating microRNA-125b-5p. Brain Res. Bull..

[91] Chen K.-P., Hua K.-F., Tsai F.-T., Lin T.-Y., Cheng C.-Y., Yang D.-I., Hsu H.-T., Ju T.-C. (2022). A Selective Inhibitor of the NLRP3 Inflammasome as a Potential Therapeutic Approach for Neuroprotection in a Transgenic Mouse Model of Hunting-ton’s Disease. J. Neuroinflammation.

[92] Barry J., Bui M.T., Levine M.S., Cepeda C. (2021). Synaptic pathology in Huntington’s disease: Beyond the corticostriatal pathway. Neurobiol. Dis..

[93] Vonsattel J.P.G., Keller C., Amaya M.D.P. (2008). Neuropathology of Huntington’s Disease. Handb. Clin. Neurol..

[94] Shimojo M. (2008). Huntingtin Regulates RE1-Silencing Transcription Factor/Neuron-Restrictive Silencer Factor (REST/NRSF) Nu-clear Trafficking Indirectly through a Complex with REST/NRSF-Interacting LIM Domain Protein (RILP) and Dynactin P150Glued. J. Biol. Chem..

[95] Zuccato C., Valenza M., Cattaneo E. (2010). Molecular Mechanisms and Potential Therapeutical Targets in Huntington’s Disease. Physiol. Rev..

[96] Samadi P., Boutet A., Rymar V.V., Rawal K., Maheux J., Kvann J.-C., Tomaszewski M., Beaubien F., Cloutier J.F., Levesque D. (2012). Relationship between BDNF expression in major striatal afferents, striatum morphology and motor behavior in the R6/2 mouse model of Huntington’s disease. Genes Brain Behav..

[97] Ma Q., Yang J., Li T., Milner T.A., Hempstead B.L. (2015). Selective reduction of striatal mature BDNF without induction of proBDNF in the zQ175 mouse model of Huntington’s disease. Neurobiol. Dis..

[98] Silva A., Naia L., Dominguez A., Ribeiro M., Rodrigues J., Vieira O.V., Lessmann V., Rego A.C. (2015). Overexpression of BDNF and Full-Length TrkB Receptor Ameliorate Striatal Neural Survival in Huntington’s Disease. Neurodegener. Dis..

[99] Nguyen K.Q., Rymar V.V., Sadikot A.F. (2016). Impaired TrkB Signaling Underlies Reduced BDNF-Mediated Trophic Support of Striatal Neurons in the R6/2 Mouse Model of Huntington’s Disease. Front. Cell. Neurosci..

[100] Yu C., Li C.H., Chen S., Yoo H., Qin X., Park H. (2018). Decreased BDNF Release in Cortical Neurons of a Knock-in Mouse Model of Huntington’s Disease. Sci. Rep..

[101] Griffioen K.J., Wan R., Brown T.R., Okun E., Camandola S., Mughal M.R., Phillips T.M., Mattson M.P. (2011). Aberrant heart rate and brainstem brain-derived neurotrophic factor (BDNF) signaling in a mouse model of Huntington’s disease. Neurobiol. Aging.

[102] Müller S. (2014). In silico analysis of regulatory networks underlines the role of miR-10b-5p and its target BDNF in huntington’s disease. Transl. Neurodegener..

[103] Betti L., Palego L., Unti E., Mazzucchi S., Kiferle L., Palermo G., Bonuccelli U., Giannaccini G., Ceravolo R. (2018). Brain-Derived Neurotrophic Factor (BDNF) and Serotonin Transporter (SERT) in Platelets of Patients with Mild Huntington’s Disease: Relationships with Social Cognition Symptoms. Arch. Ital. Biol..

[104] Gutierrez A., Corey-Bloom J., Thomas E.A., Desplats P. (2020). Evaluation of Biochemical and Epigenetic Measures of Peripheral Brain-Derived Neurotrophic Factor (BDNF) as a Biomarker in Huntington’s Disease Patients. Front. Mol. Neurosci..

[105] Plinta K., Plewka A., Pawlicki K., Zmarzły N., Wójcik-Pędziwiatr M., Rudziński M., Krzak-Kubica A., Doręgowska-Stachera M., Rudzińska-Bar M. (2021). The Utility of BDNF Detection in Assessing Severity of Huntington’s Disease. J. Clin. Med..

[106] Park H. (2018). Cortical Axonal Secretion of BDNF in the Striatum Is Disrupted in the Mutant-huntingtin Knock-in Mouse Model of Huntington’s Disease. Exp. Neurobiol..

[107] Zhou Z., Zhong S., Zhang R., Kang K., Zhang X., Xu Y., Zhao C., Zhao M. (2021). Functional analysis of brain derived neurotrophic factor (BDNF) in Huntington’s disease. Aging.

[108] Martire A., Pepponi R., Domenici M.R., Ferrante A., Chiodi V., Popoli P. (2013). BDNF prevents NMDA-induced toxicity in models of Huntington’s disease: The effects are genotype specific and adenosine A2A receptor is involved. J. Neurochem..

[109] Smail S., Bahga D., McDole B., Guthrie K. (2016). Increased Olfactory Bulb BDNF Expression Does Not Rescue Deficits in Olfactory Neurogenesis in the Huntington’s Disease R6/2 Mouse. Chem. Senses.

[110] Torres-Cruz F.M., Mendoza E., Vivar-Cortés I.C., García-Sierra F., Hernández-Echeagaray E. (2019). Do BDNF and NT-4/5 exert synergistic or occlusive effects on corticostriatal transmission in a male mouse model of Huntington’s disease?. J. Neurosci. Res..

[111] Kiernan M.C., Vucic S., Cheah B.C., Turner M.R., Eisen A., Hardiman O., Burrell J.R., Zoing M.C. (2011). Amyotrophic Lateral Sclerosis. Lancet.

[112] Dhasmana S., Dhasmana A., Narula A.S., Jaggi M., Yallapu M.M., Chauhan S.C. (2021). The panoramic view of amyotrophic lateral sclerosis: A fatal intricate neurological disorder. Life Sci..

[113] Marin B., Boumédiene F., Logroscino G., Couratier P., Babron M.-C., Leutenegger A.-L., Copetti M., Preux P.-M., Beghi E. (2017). Variation in worldwide incidence of amyotrophic lateral sclerosis: A meta-analysis. Int. J. Epidemiol..

[114] Rosen D.R., Siddique T., Patterson D., Figlewicz D.A., Sapp P., Hentati A., Donaldson D., Goto J., O’Regan J.P., Deng H.-X. (1993). Mutations in Cu/Zn superoxide dismutase gene are associated with familial amyotrophic lateral sclerosis. Nature.

[115] Hayashi Y., Homma K., Ichijo H. (2016). SOD1 in neurotoxicity and its controversial roles in SOD1 mutation-negative ALS. Adv. Biol. Regul..

[116] Xu L., Tian D., Li J., Chen L., Tang L., Fan D. (2017). The Analysis of Two BDNF Polymorphisms G196A/C270T in Chinese Sporadic Amyotrophic Lateral Sclerosis. Front. Aging Neurosci..

[117] Tremolizzo L., Pellegrini A., Conti E., Arosio A., Gerardi F., Lunetta C., Magni P., Appollonio I., Ferrarese C. (2016). BDNF Serum Levels with Respect to Multidimensional Assessment in Amyotrophic Lateral Sclerosis. Neurodegener. Dis..

[118] Yamazaki T. (2010). Immunohistochemical Studies of Brain-Derived Neurotrophic Factor in Skin of Patients with Amyotrophic Lateral Sclerosis. Teikyo Med. J..

[119] Hurtado E., Cilleros V., Nadal L., Simó A., Obis T., Garcia N., Santafe M., Tomàs M., Halievski K., Jordan C.L. (2017). Muscle Contraction Regulates BDNF/TrkB Signaling to Modulate Synaptic Function through Presynaptic cPKCα and cPKCβI. Front. Mol. Neurosci..

[120] Lanuza M.A., Just-Borràs L., Hurtado E., Cilleros-Mañé V., Tomàs M., Garcia N., Tomàs J. (2019). The Impact of Kinases in Amyotrophic Lateral Sclerosis at the Neuromuscular Synapse: Insights into BDNF/TrkB and PKC Signaling. Cells.

[121] Just-Borràs L., Hurtado E., Cilleros-Mañé V., Biondi O., Charbonnier F., Tomàs M., Garcia N., Lanuza M.A., Tomàs J. (2019). Overview of Impaired BDNF Signaling, Their Coupled Downstream Serine-Threonine Kinases and SNARE/SM Complex in the Neuromuscular Junction of the Amyotrophic Lateral Sclerosis Model SOD1-G93A Mice. Mol. Neurobiol..

[122] Yanpallewar S.U., Barrick C.A., Buckley H., Becker J., Tessarollo L. (2012). Deletion of the BDNF Truncated Receptor TrkB.T1 Delays Disease Onset in a Mouse Model of Amyotrophic Lateral Sclerosis. PLoS ONE.

[123] Yanpallewar S., Fulgenzi G., Tomassoni-Ardori F., Barrick C., Tessarollo L. (2020). Delayed onset of inherited ALS by deletion of the BDNF receptor TrkB.T1 is non-cell autonomous. Exp. Neurol..

[124] Park S., Kim H.-T., Yun S., Kim I.-S., Lee J., Lee I.-S., Park K.I. (2009). Growth factor-expressing human neural progenitor cell grafts protect motor neurons but do not ameliorate motor performance and survival in ALS mice. Exp. Mol. Med..

[125] (1999). BDNF Study Group A Controlled Trial of Recombinant Methionyl Human BDNF in ALS: The BDNF Study Group (Phase III). Neurology.

[126] Ochs G., Penn R.D., York M., Giess R., Beck M., Tonn J., Haigh J., Malta E., Traub M., Sendtner M. (2000). A phase I/II trial of recombinant methionyl human brain derived neurotrophic factor administered by intrathecal infusion to patients with amyotrophic lateral sclerosis. Amyotroph. Lateral Scler. Other Mot. Neuron Disord..

[127] Kalra S., Genge A., Arnold D.L. (2003). A prospective, randomized, placebo-controlled evaluation of corticoneuronal response to intrathecal BDNF therapy in ALS using magnetic resonance spectroscopy: Feasibility and results. Amyotroph. Lateral Scler. Other Mot. Neuron Disord..

[128] Beck M., Flachenecker P., Magnus T., Giess R., Reiners K., Toyka K.V., Naumann M. (2005). Autonomic dysfunction in ALS: A preliminary study on the effects of intrathecal BDNF. Amyotroph. Lateral Scler..

[129] Calvo A.C., Moreno-Igoa M., Mancuso R., Manzano R., Oliván S., Muñoz M.J., Penas C., Zaragoza P., Navarro X., Osta R. (2011). Lack of a synergistic effect of a non-viral ALS gene therapy based on BDNF and a TTC fusion molecule. Orphanet J. Rare Dis..

[130] Shruthi S., Sumitha R., Varghese A.M., Ashok S., Chandrasekhar Sagar B.K., Sathyaprabha T.N., Nalini A., Kramer B.W., Raju T.R., Vijayalakshmi K. (2016). Brain-Derived Neurotrophic Factor Facilitates Functional Recovery from ALS-Cerebral Spinal Fluid-Induced Neurodegenerative Changes in the NSC-34 Motor Neuron Cell Line. Neuro Degener. Dis..

[131] Wang J., Hu W., Feng Z., Feng M. (2020). BDNF-overexpressing human umbilical cord mesenchymal stem cell-derived motor neurons improve motor function and prolong survival in amyotrophic lateral sclerosis mice. Neurol. Res..

[132] Jaiswal M.K. (2018). Riluzole and edaravone: A tale of two amyotrophic lateral sclerosis drugs. Med. Res. Rev..

[133] Turner B.J., Murray S.S., Piccenna L.G., Lopes E.C., Kilpatrick T.J., Cheema S.S. (2004). Effect of p75 neurotrophin receptor antagonist on disease progression in transgenic amyotrophic lateral sclerosis mice. J. Neurosci. Res..

[134] Mellesmoen A., Sheeler C., Ferro A., Rainwater O., Cvetanovic M. (2019). Brain Derived Neurotrophic Factor (BDNF) Delays Onset of Pathogenesis in Transgenic Mouse Model of Spinocerebellar Ataxia Type 1 (SCA1). Front. Cell. Neurosci..

[135] Sheeler C., Rosa J.-G., Borgenheimer E., Mellesmoen A., Rainwater O., Cvetanovic M. (2021). Post-symptomatic Delivery of Brain-Derived Neurotrophic Factor (BDNF) Ameliorates Spinocerebellar Ataxia Type 1 (SCA1) Pathogenesis. Cerebellum.

[136] Takahashi M., Ishikawa K., Sato N., Obayashi M., Niimi Y., Ishiguro T., Yamada M., Toyoshima Y., Takahashi H., Kato T. (2012). Reduced brain-derived neurotrophic factor (BDNF) mRNA expression and presence of BDNF-immunoreactive granules in the spinocerebellar ataxia type 6 (SCA6) cerebellum. Neuropathology.

[137] Misiorek J.O., Schreiber A.M., Urbanek-Trzeciak M.O., Jazurek-Ciesiołka M., Hauser L.A., Lynch D.R., Napierala J.S., Napierala M. (2020). A Comprehensive Transcriptome Analysis Identifies FXN and BDNF as Novel Targets of miRNAs in Friedreich’s Ataxia Patients. Mol. Neurobiol..

[138] Islas-Hernandez A., Aguilar-Talamantes H.S., Bertado-Cortes B., Mejia-Delcastillo G.D.J., Carrera-Pineda R., Cuevas-Garcia C.F., Garcia-Delatorre P. (2018). BDNF and Tau as biomarkers of severity in multiple sclerosis. Biomark. Med..

[139] Oraby M.I., El Masry H.A., El Shafy S.S.A., Galil E.M.A. (2021). Serum level of brain-derived neurotrophic factor in patients with relapsing–remitting multiple sclerosis: A potential biomarker for disease activity. Egypt. J. Neurol. Psychiatry Neurosurg..

[140] Naegelin Y., Saeuberli K., Schaedelin S., Dingsdale H., Magon S., Baranzini S., Amann M., Parmar K., Tsagkas C., Calabrese P. (2020). Levels of brain-derived neurotrophic factor in patients with multiple sclerosis. Ann. Clin. Transl. Neurol..

[141] Nociti V., Santoro M., Quaranta D., Losavio F.A., De Fino C., Giordano R., Palomba N., Rossini P.M., Guerini F.R., Clerici M. (2018). BDNF rs6265 polymorphism methylation in Multiple Sclerosis: A possible marker of disease progression. PLoS ONE.

[142] Dolcetti E., Bruno A., Azzolini F., Gilio L., Moscatelli A., De Vito F., Pavone L., Iezzi E., Gambardella S., Giardina E. (2022). The BDNF Val66Met Polymorphism (rs6265) Modulates Inflammation and Neurodegeneration in the Early Phases of Multiple Sclerosis. Genes.

[143] De Meo E., Portaccio E., Prestipino E., Nacmias B., Bagnoli S., Razzolini L., Pastò L., Niccolai C., Goretti B., Bellinvia A. (2021). Effect of BDNF Val66Met polymorphism on hippocampal subfields in multiple sclerosis patients. Mol. Psychiatry.

[144] Giordano A., Clarelli F., Cannizzaro M., Mascia E., Santoro S., Sorosina M., Ferrè L., Leocani L., Esposito F. (2022). BDNF Val66Met Polymorphism Is Associated with Motor Recovery After Rehabilitation in Progressive Multiple Sclerosis Patients. Front. Neurol..

[145] Portaccio E., Bellinvia A., Prestipino E., Nacmias B., Bagnoli S., Razzolini L., Pastò L., Niccolai C., Goretti B., Fonderico M. (2021). The Brain-Derived Neurotrophic Factor Val66Met Polymorphism Can Protect Against Cognitive Impairment in Multiple Sclerosis. Front. Neurol..

[146] Konukoglu D., Andican G., Fırtına S., Erkol G., Kurt A. (2012). Serum brain-derived neurotrophic factor, nerve growth factor and neurotrophin-3 levels in dementia. Acta Neurol. Belg..

[147] Weinstein G., Beiser A., Choi S.H., Preis S.R., Chen T.C., Vorgas D., Au R., Pikula A., Wolf P.A., DeStefano A.L. (2014). Serum Brain-Derived Neurotrophic Factor and the Risk for Dementia: The Framingham Heart Study. JAMA Neurol..

[148] Huey E.D., Fremont R., Manoochehri M., Gazes Y., Lee S., Cosentino S., Tierney M., Wassermann E.M., Momeni P., Grafman J. (2020). Effect of Functional BDNF and COMT Polymorphisms on Symptoms and Regional Brain Volume in Frontotemporal Dementia and Corticobasal Syndrome. J. Neuropsychiatry Clin. Neurosci..

[149] Huang Y., Li Z., Nan G. (2017). Effect of hippocampal L-NBP on BDNF and TrkB expression and neurological function of vascular dementia rats. Mol. Med. Rep..

[150] Kim Y., Kim Y.-J. (2020). Effect of Obesity on Cognitive Impairment in Vascular Dementia Rat Model via BDNF-ERK-CREB Pathway. Biol. Res. Nurs..

[151] Forlenza O.V., Diniz B., Teixeira A.L., Ojopi E.B., Talib L.L., Mendonça V.A., Izzo G., Gattaz W.F. (2010). Effect of brain-derived neurotrophic factor Val66Met polymorphism and serum levels on the progression of mild cognitive impairment. World, J. Biol. Psychiatry.

[152] Cechova K., Andel R., Angelucci F., Chmatalova Z., Markova H., Laczó J., Vyhnalek M., Matoska V., Kaplan V., Nedelska Z. (2020). Impact of APOE and BDNF Val66Met Gene Polymorphisms on Cognitive Functions in Patients with Amnestic Mild Cognitive Impairment. J. Alzheimer’s Dis..

[153] Ng T.K.S., Coughlan C., Heyn P.C., Tagawa A., Carollo J.J., Kua E.H., Mahendran R. (2021). Increased plasma brain-derived neurotrophic factor (BDNF) as a potential biomarker for and compensatory mechanism in mild cognitive impairment: A case-control study. Aging.

[154] Balietti M., Giuli C., Casoli T., Fabbietti P., Conti F. (2020). Is Blood Brain-Derived Neurotrophic Factor a Useful Biomarker to Monitor Mild Cognitive Impairment Patients?. Rejuvenation Res..

[155] Shimada H., Makizako H., Doi T., Yoshida D., Tsutsumimoto K., Anan Y., Uemura K., Lee S., Park H., Suzuki T. (2014). A Large, Cross-Sectional Observational Study of Serum BDNF, Cognitive Function, and Mild Cognitive Impairment in the Elderly. Front. Aging Neurosci..

